# Social Media as a Research Tool (SMaaRT) for Risky Behavior Analytics: Methodological Review

**DOI:** 10.2196/21660

**Published:** 2020-11-30

**Authors:** Tavleen Singh, Kirk Roberts, Trevor Cohen, Nathan Cobb, Jing Wang, Kayo Fujimoto, Sahiti Myneni

**Affiliations:** 1 School of Biomedical Informatics The University of Texas Health Science Center Houston, TX United States; 2 Biomedical Informatics and Medical Education University of Washington Seattle, WA United States; 3 Georgetown University Medical Center Washington, DC United States; 4 School of Nursing The University of Texas Health Science Center San Antonio, TX United States; 5 School of Public Health The University of Texas Health Science Center Houston, TX United States

**Keywords:** social media, infodemiology, infoveillance, online health communities, risky health behaviors, data mining, machine learning, natural language processing, text mining

## Abstract

**Background:**

Modifiable risky health behaviors, such as tobacco use, excessive alcohol use, being overweight, lack of physical activity, and unhealthy eating habits, are some of the major factors for developing chronic health conditions. Social media platforms have become indispensable means of communication in the digital era. They provide an opportunity for individuals to express themselves, as well as share their health-related concerns with peers and health care providers, with respect to risky behaviors. Such peer interactions can be utilized as valuable data sources to better understand inter-and intrapersonal psychosocial mediators and the mechanisms of social influence that drive behavior change.

**Objective:**

The objective of this review is to summarize computational and quantitative techniques facilitating the analysis of data generated through peer interactions pertaining to risky health behaviors on social media platforms.

**Methods:**

We performed a systematic review of the literature in September 2020 by searching three databases—PubMed, Web of Science, and Scopus—using relevant keywords, such as “social media,” “online health communities,” “machine learning,” “data mining,” etc. The reporting of the studies was directed by the PRISMA (Preferred Reporting Items for Systematic Reviews and Meta-Analyses) guidelines. Two reviewers independently assessed the eligibility of studies based on the inclusion and exclusion criteria. We extracted the required information from the selected studies.

**Results:**

The initial search returned a total of 1554 studies, and after careful analysis of titles, abstracts, and full texts, a total of 64 studies were included in this review. We extracted the following key characteristics from all of the studies: social media platform used for conducting the study, risky health behavior studied, the number of posts analyzed, study focus, key methodological functions and tools used for data analysis, evaluation metrics used, and summary of the key findings. The most commonly used social media platform was Twitter, followed by Facebook, QuitNet, and Reddit. The most commonly studied risky health behavior was nicotine use, followed by drug or substance abuse and alcohol use. Various supervised and unsupervised machine learning approaches were used for analyzing textual data generated from online peer interactions. Few studies utilized deep learning methods for analyzing textual data as well as image or video data. Social network analysis was also performed, as reported in some studies.

**Conclusions:**

Our review consolidates the methodological underpinnings for analyzing risky health behaviors and has enhanced our understanding of how social media can be leveraged for nuanced behavioral modeling and representation. The knowledge gained from our review can serve as a foundational component for the development of persuasive health communication and effective behavior modification technologies aimed at the individual and population levels.

## Introduction

Modifiable risky health behaviors, such as tobacco use, excessive alcohol use, being overweight, lack of physical activity, and unhealthy eating habits, are some of the major factors for developing chronic health conditions [1]. Chronic health conditions, such as cancer and heart disease, lead to approximately 1.5 million deaths per year in the United States [2]. These chronic health conditions together with diabetes are also responsible for nearly US $3.5 trillion in annual economic costs; hence, it becomes crucial to prevent and/or efficiently manage such conditions [2]. Behavior modification is pivotal for managing chronic health conditions, and a range of psychological and social processes have been shown to influence the engagement of an individual in the adoption of positive healthy behaviors [3,4]. Traditionally, the methods used for measuring and studying health-related behaviors in populations include telephone or internet-based surveys [5], motivational interviews [6], commercial wearables and smartphone apps [7], and ecological momentary assessment [8].

Recently, social media has emerged as a viable platform for studying and analyzing health-related behaviors and promoting behavior change [9]. The field of infodemiology [10] examines the determinants and distribution of health information in the electronic medium (eg, social media and internet) for public health purposes: preventing diseases via predictive modeling [11-13], informing policy regulations [14], assessing the quality of health information on websites [15], and analyzing the health-related behaviors of individuals [16-18]. The recent COVID-19 pandemic has also shown how analyzing communication on such platforms can provide insights into the attitudes and behaviors of individuals as well as health care providers [19,20].

Social media, through its various mobile and web-based technologies, provides interactive platforms for individuals and communities to share, create, modify, and discuss content in the form of ideas, messages, or information [21]. In recent years, the penetration of social media platforms has increased in all spheres of life. According to the Global Digital Report of 2019, there are about 3.5 billion active social media users throughout the world, with Facebook being the most dominant social networking website. More than two-thirds of the world’s population use a mobile device, mostly a smartphone. Powered by these connected devices, many older adults as well as teenagers have also started incorporating social media into their daily routines [22].

Consequently, social media has become an important part of the public health landscape, given that these platforms are increasingly being used by health care consumers for gaining knowledge on a variety of health-related topics as well as for interacting with their peers and health care providers to garner social support, mostly informational and emotional in nature [23,24]. These platforms are widely used by health care consumers to (1) meet their health-related goals [25] and (2) adopt positive health behaviors [26,27]. Research has shown that an individual is more likely to comply with health-related goals and adhere to preventive practices provided their social ties also engage in similar behaviors [28,29]. The major advantages of using such platforms over standard approaches for studying and analyzing health promotion and behavior change include their ability to reach a wider and less accessible audience, cost-effective recruitment of participants for research, and their round-the-clock accessibility via mobile and web-based connections [30]. These platforms can leverage group norms; thus, behavior change interventions implemented through these platforms have the potential to make a significant impact through widespread diffusion of preventive programs to meet the needs of individuals, communities, and populations.

These online platforms can be broadly classified into two major categories: (1) open social media platforms (eg, Facebook, Twitter, and Reddit), which are generic platforms used for networking, information sharing, and collaboration, and (2) intentionally designed health-related social media platforms (eg, QuitNet [31] and BecomeAnEX.org [32]), which focus on providing health-specific support to its members. Even though open social media platforms provide opportunities for large-scale inferences about behaviors of individuals, they still lack in providing context-specific interactional observations, for which we need to turn to intentionally designed social media platforms [33]. Depending on whether or not a social media platform has a specific focus on health topics, the environmental factors affecting an individual’s attempt to sustain positive health changes can greatly vary, thus affecting contextual granularities that inform the accuracy and reliability of computational and quantitative data modeling approaches. Despite these differences, the universal presence of these platforms has led to the generation of invaluable and large data sets in the form of electronic traces of peer interactions in the form of text, images, or videos (eg, traditional forums like Facebook and YouTube). These data sets capture the attitudes and behaviors of individuals in near real time and in natural settings as compared to conventional settings, which involve the presence of a researcher and are prone to instrument bias [34]. The analysis of such data sets provides us with an opportunity to understand the individualistic as well as environmental factors underlying behavior change, which can eventually guide the design and development of network interventions for health-related behavior change [35-37].

Traditional methods of qualitative data analysis are not conducive to analyzing large amounts of data generated by social media platforms. Recent advances in automated text analysis provide us with suitable methods for analyzing digital content generated from social media platforms. The latest review highlights the breakthroughs in computational technologies that are currently being applied to the field of health care in the form of digitized data acquisition, machine learning (ML) techniques, and computing infrastructure [38]. In addition to advances in predictive analytics and combinatorial forces from mobile computing and the internet, participatory social media has resulted in rich, just-in-time data that can be leveraged to conduct digital phenotyping of health consumer engagement in self-management of risky health behaviors.

The objective of this review is to summarize computational and quantitative approaches that highlight the potential of using social media as a research tool (SMaaRT) to understand the patterns of inter- and intrapersonal psychosocial factors associated with the prevention and management of risky health behaviors. These methodologies can provide a comprehensive understanding of the most common practices, their utility, limitations, and resulting inferences, thus providing health researchers with capabilities to better describe health behaviors at scale. The enhanced understanding from these secondary analyses can ultimately be infused into the design processes of effective behavioral interventions through the translation of data-driven insights into practical public health solutions via scalable techniques, such as tailored messaging and persuasive environment design.

## Methods

### Overview

We conducted a systematic review of the literature to summarize the computational and quantitative methods for analyzing social media data that have been used to study risky health behaviors. We followed the guidelines outlined by PRISMA (Preferred Reporting Items for Systematic Reviews and Meta-Analyses) [39] to retrieve relevant studies.

### Literature Search Strategy

We searched the literature in September 2020, collecting studies published between 2011 and September 11, 2020. We searched three different databases—PubMed, Web of Science, and Scopus—using a specific set of keywords. Our search keywords lie at the intersection of two key clusters: social media and ML. We also included Medical Subject Headings (MeSH) for relevant keywords to ensure our search was as inclusive as possible. The search was conducted using the following query: (“Social Media” [MeSH] OR “social media” OR “Online Health Community” OR “Online Health Communities” OR “Online Social Network” OR “Online Social Networks” OR “peer to peer” OR “Peer Influence” [MeSH]) AND (“Machine Learning” [MeSH] OR “machine learning” OR “text mining” OR “Natural Language Processing” [MeSH] OR “natural language processing” OR “Data Mining” [MeSH] OR “data mining” OR “network models”). In addition, we also examined the reference lists of studies that met our inclusion criteria for any additional sources.

### Inclusion and Exclusion Criteria

The inclusion and exclusion criteria to determine eligibility of studies for the review are listed in Textbox 1.

**Eligibility criteria for the studies.**

Inclusion criteria:

1. Studies conducted original research that was published in a peer-reviewed journal.
2. Studies used English language–based social media platforms (ie, the language of generated content is in the English language).
3. Studies conducted data analysis at scale using computational or quantitative methods like machine learning techniques, network modeling, and/or visualization techniques.
4. Studies focused on risky health behaviors, or related attitudes or beliefs, of the patients or health consumers such as nicotine use, alcohol use, drug or substance abuse, physical activity or inactivity patterns, or obesity-related behaviors.
5. Studies focused primarily on analyzing textual content from online social media platforms (eg, YouTube comments instead of YouTube videos).

Exclusion criteria:

1. Studies described the use of social media platforms for other purposes (eg, recruitment and data collection).
2. Studies focused on health care providers instead of patients or health consumers.
3. Studies focused on behaviors unrelated to health.

### Data Extraction

Two authors (TS and SM) independently assessed the retrieved studies against the inclusion criteria in two stages. In the first stage, the authors reviewed the titles and abstracts of all the retrieved studies for their inclusion in full-text screening. In the second stage, the authors performed the full-text screening of the relevant studies identified from the first stage for final inclusion in this review. Disagreements were resolved through discussion between the two authors. The interrater agreement, Cohen κ, was calculated at both stages. After screening the studies that met our inclusion criteria, we extracted the relevant data from the main text, which included the following:

1. Risky health behavior studied, such as nicotine use, alcohol use, drug or substance abuse, physical activity or inactivity patterns, obesity-related behaviors, etc.
2. Social media platform used for the study, whether it was an open social network, such as Twitter or Facebook, or a disease-specific social network, such as QuitNet (ie, smoking cessation).
3. Number of posts: total number of posts used for analysis and number of posts used for manual annotations.
4. Study focus: what were the underlying aims of the study for analyzing risky health behaviors?
5. Key methodological functions and tools; for example, topic modeling (ie, function) was performed using latent Dirichlet allocation (LDA) (ie, method).
6. Evaluation metrics used by the study (eg, precision, recall, and F1 score).
7. Key findings of the study: results obtained after analyzing the data generated from online peer interactions.

## Results

### Overview

**Figure 1:**
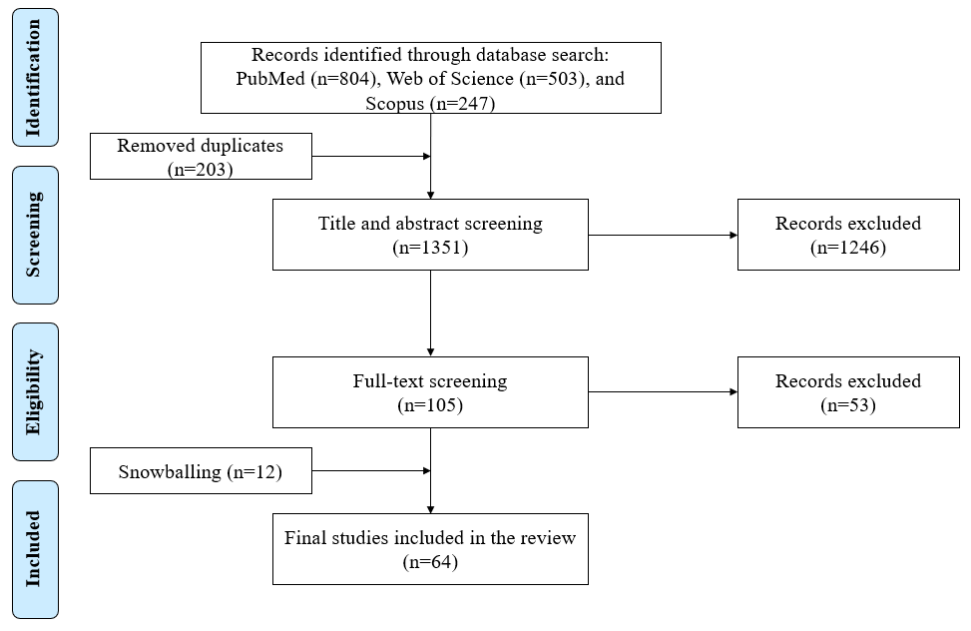
PRISMA (Preferred Reporting Items for Systematic Reviews and Meta-Analyses) diagram for study selection.

The initial search resulted in a total of 1554 studies. From these, we removed 203 studies because of duplication. In the first stage, we reviewed the titles and abstracts of the remaining studies to ensure that they met the inclusion and exclusion criteria for further thorough analysis. The interrater agreement at the first stage was 81.37%. After resolving disagreements through discussion, we initially excluded 1246 studies that did not meet the inclusion criteria and included the remaining 105 studies for full-text screening in the second stage. The interrater agreement at the second stage was 83.50%. A total of 52 studies meeting the inclusion criteria were included in the review. We further identified 12 additional studies through the snowballing technique that were also included in this review. Thus, a total of 64 studies [40-103] were included in the final review. Of the studies reviewed, 55 (86%) studies were published from 2016 onward [40-61,68-95,97,98,100-102], while only 9 (14%) studies were published between 2013 and 2015 [62-67,96,99,103]. None of the studies were published before 2013. Figure 1 shows the PRISMA diagram highlighting the overall process of selecting the final studies for the review.

The results of our review showed that the focus of social media analysis has been on a variety of risky health behaviors, including nicotine use, alcohol use, drug abuse, physical activity patterns, and obesity-related behaviors. Social media platforms have been widely used for secondary data analysis as well as for follow-up analysis of data generated from active interventions or campaigns conducted using such platforms. Multiple computational and quantitative functions and tools were utilized for analyzing the data generated from online peer interactions on social media platforms. A detailed exposition of our results is included in Multimedia Appendix 1, which shows the key characteristics of the selected studies grouped by risky health behaviors and then ordered by year published.

In the following sections, we aggregate the results of our review to highlight the usage patterns of various social media platforms for secondary analysis purposes, the prevalence of risky health behaviors studied on these platforms, and the methodological tools and functions used to understand these behaviors.

### Social Media Platforms

Table 1 [40-103] highlights the social media platforms used for analyzing risky health behaviors. Twitter (39/64, 61%) appeared to be the most widely utilized social media platform for analyzing online peer interactions regarding risky health behaviors, followed by Facebook (6/64, 9%), QuitNet (5/64, 8%), Reddit (5/64, 8%), BecomeAnEx.org (3/64, 5%), Instagram (2/64, 3%), Cancer Survivors Network (1/64, 2%), Hello Sunday Morning blog (1/64, 2%), patient.info/forums (1/64, 2%), and a peer-to-peer online discussion forum, which is part of a smartphone app called Addiction–Comprehensive Health Enhancement Support System (A-CHESS) (1/64, 2%). Out of 64 studies, 1 (2%) analyzed the data from three online forums: Vapor Talk, Hookah Forum, and Stopsmoking subreddit [62]. A total of 80% (51/64) of the studies utilized open social media platforms, such as Twitter, Facebook, Instagram, and Reddit [40-44,47-54,58-61,63,66-83,85,87,88,92-103], while the remaining 20% (13/64) of the studies utilized specific health-related online social networks, such as QuitNet, BecomeAnEX.org, Cancer Survivors Network, patient.info/forums, Hello Sunday Morning blog, and A-CHESS online discussion forum [45,46,55-57,62,64,65,84,86,89-91].

Most of the studies that used Twitter as their data source relied on Twitter application programming interfaces (APIs) for extracting the data. The majority of these studies utilized streaming APIs, which provide a push of the subset of data in near real time [47,50,51,59,61,70,74,78-81,92,94,95], and some of these studies also used search APIs, which provide access to the data set that consists of tweets that have already occurred in the past [68,76,82,98,99]. Some studies also used Twitter’s data provider called Gnip [54,59,60,63,92], which guarantees access to all the tweets that match the researcher’s criteria. Some studies did not indicate which specific kind of API was used for accessing Twitter’s data [40,41,48,66,73,77,88,100,102]. For Reddit, the data were extracted using the following techniques: (1) the use of Pushshift, which is a publicly available archive of Reddit submissions [42], (2) the data set was downloaded using a web crawler called Wget [62], (3) the use of Python Reddit API Wrapper [97], (4) the data set was released from the Reddit member [101], and (5) the use of Reddit’s official API [103]. The data from Facebook were extracted using either Facebook’s API and the Facebook platform’s Python software development kit [87] or by using the extraction feature in NVivo (QSR International) [71]. A similar approach was used for extracting data using Instagram’s API [44,72].

**Table 1:** Social media platforms used by various studies.

Social media platforms	Number of studies (N=64), n (%)^a^	Study references
Twitter	39 (61)	[40,41,43,47, 48,50-52, 54,58-61, 63,66-70,73-83, 88,92,94-96,98-100,102]
Facebook	6 (9)	[49,53,71,85,87,93]
QuitNet	5 (8)	[45,55,56,64,65]
Reddit	5 (8)	[42,62,97,101,103]
BecomeAnEX.org	3 (5)	[46,86,91]
Instagram	2 (3)	[44,72]
Hello Sunday Morning blog	1 (2)	[90]
A-CHESS^b^ (online discussion forum)	1 (2)	[89]
Cancer Survivors Network	1 (2)	[57]
Patient.info/forums	1 (2)	[84]
Vapor Talk, Hookah Forum, and Stopsmoking subreddit	1 (2)	[62]

^a^Percentages do not add up to 100% due to rounding and one study that used multiple social media platforms.

^b^A-CHESS: Addiction–Comprehensive Health Enhancement Support System.

### Risky Health Behaviors

Table 2 [40-103] highlights the risky health behaviors studied and the associated social media platforms leveraged for conducting the study. The most commonly studied risky health behavior on social media platforms was related to the use of nicotine products, with a total of 28 out of 64 (44%) studies [40-67] focusing on behaviors related to smoking, e-cigarettes, little cigars, etc. Twitter (16/64, 25%) was widely used for analyzing such behaviors, followed by QuitNet (5/64, 8%), Facebook (2/64, 3%), Reddit (1/64, 2%), Instagram (1/64, 2%), Cancer Survivors Network (1/64, 2%), BecomeAnEX.org (1/64, 2%), and Vapor Talk, Hookah Forum, and Stopsmoking subreddit (1/64, 2%). The majority of these studies were focused on analyzing members’ behavior or sentiment toward smoking products, such as e-cigarettes [42,49,50,52,54,58,59,61-63], hookah products [43,47,51,62], JUUL or vaping [40,41,44], and cigars [60], or analyzing sentiments toward smoking in general [67]. Out of 64 studies, 2 (3%) focused primarily on social network analysis: one to understand how the structure of social networks influence the smoking behaviors of the members of the community [53], and the other to understand the reach of an antismoking campaign targeting young individuals [48]. Other studies focused on (1) analyzing member-generated content to derive common themes or topics of discussions among peers [57,64-66], (2) characterizing behavioral transitions during smoking cessation [45], (3) studying temporal trends of peer interactions to gain insights into factors underlying smoking cessation behavior change [55,56], and (4) predicting smoking status [46].

Drug or substance abuse was another commonly discussed risky health behavior on social media platforms, with a total of 14 out of 64 (22%) studies discussing the topic [68-81]. Twitter (12/64, 19%) again was the most popular platform for studying drug or substance abuse behaviors, followed by Instagram (1/64, 2%) and Facebook (1/64, 2%). The focus areas for these studies included prescription drug abuse [68,70,78,81], opioid misuse [74-77], cannabis and synthetic cannabinoid use [80], and substance or drug abuse [69,71-73]. One study analyzed multiple behaviors related to substance abuse, which included alcohol, smoking, and drug use [79].

Out of 64 studies, 12 (19%) explored the alcohol usage patterns and abstinence behaviors among members of online health communities [82-93]. Some of these studies (1) conducted a thematic analysis of alcohol-related content generated from an online smoking cessation community [86,91], (2) focused on analyzing trends of alcohol use behavioral stages [92], (3) analyzed binge-drinking behaviors [82,83,87], (4) focused on extracting topics and sentiments related to alcohol use [84,85,93], and (5) focused on predicting future relapse or recovery alcoholism [88,89]. One study analyzed the content of a blog that encouraged its members to stop drinking for a specific period of time and discuss their progress with their peers [90]. The distribution of platforms used for analyzing alcohol use behaviors was quite variable (see Table 2 [40-103]).

Out of 64 studies, 3 (5%) explored the patterns and types of physical activity engagement among members of the community [94-96]. All of these studies were conducted using Twitter as their source of data. Out of 64 studies, 3 (5%) analyzed topics and themes related to obesity-related behaviors [97-99] using social media platforms, such as Twitter and Reddit. There were 4 out of 64 (6%) studies [100-103] that studied multiple behaviors together, such as (1) analyzing obesity and physical activity–related content in order to get information about the health status of individuals [100], (2) identifying topics of discussion related to e-cigarettes and marijuana use [101], and (3) characterizing tobacco- and alcohol-related behavioral patterns [102,103]. Out of these 4 studies, 2 (50%) utilized Twitter [100,102] and 2 (50%) utilized Reddit [101,103] as their data source.

**Table 2:** Risky health behaviors and their associated social media platforms.

Risky health behaviors	Number of studies (N=64), n (%)^a^	Social media platforms and study references
Nicotine use	28 (44)	Twitter [40,41,43,47,48,50-52,54,58-61,63,66,67] QuitNet [45,55,56,64,65] Facebook [49,53] Reddit [42] Instagram [44] Cancer Survivors Network [57] BecomeAnEX.org [46] Vapor Talk, Hookah Forum, and Stopsmoking subreddit [62]
Drug and substance abuse	14 (22)	Twitter [68-70,73-81] Instagram [72] Facebook [71]
Alcohol use	12 (19)	Twitter [82,83,88,92] Facebook [85,87,93] Patient.info/forums [84] BecomeAnEX.org [86,91] A-CHESS^b^ online discussion forum [89] Hello Sunday Morning blog [90]
Physical activity	3 (5)	Twitter [94-96]
Obesity-related behaviors	3 (5)	Reddit [97] Twitter [98,99]
Multiple behaviors (ie, e-cigarettes and marijuana, smoking and drinking, and physical activity and obesity-related behaviors)	4 (6)	Twitter [100,102] Reddit [101,103]

^a^Percentages do not add up to 100 due to rounding.

^b^A-CHESS: Addiction–Comprehensive Health Enhancement Support System.

### Methodological Details and Related Tools

The methodological functions used across various studies are discussed in the following sections, as well as the specific tools used for performing those functions.

#### Computational Modeling: Feature Extraction

The most commonly extracted features were n-grams (eg, unigrams, bigrams, and trigrams) [40,44,46,47,58,59, 63,66, 67, 70,74, 75, 80-82,86, 91,92,96, 99,100,102,103]. In addition to that, some studies also made use of additional features like count vectors [41], term frequency–inverse document frequency vectors [41,63,80,82,86,87,91,92,100], language-based covariates [42], number of hashtags [44], number of hashtags containing specific strings [44], usernames [44], part of speech tags [59], sentiment scores [59,68], presence of specific terms in usernames [59], domain-specific features [46], Doc2Vec features [46], author-based features [46], thread-based features [46], user metadata features [54,82,86,92], derived behavior features (eg, unique keyword count in original tweets, unique keyword count in hashtags in original tweets, etc) [54], personal noun [68], nonmedical use terms [68], medical use terms [68], side-effect terms [68], presence of a URL [68], abuse indication terms [73-75,81], drug-slang lexicon [73,81], synonym expansion features using WordNet [73,81], word cluster features [73-75,81], features based on behavior coping styles [88], social factors [88], age [88], and image-based features [72]. Some studies used feature selection techniques, such as SelectKBest [40], information gain [66], and the chi-square test [80]. One study performed evaluation of relevant features for each classifier using a technique called SHAP (SHapley Additive exPlanations) [41].

#### Computational Modeling: Classification Techniques

##### Traditional ML Classifiers

Most of the studies utilized supervised ML classifiers for text analysis to perform either predictive modeling, behavioral stage modeling, or content analysis. The classifiers used across various studies included support vector machine (SVM) [51,54,66,67,70,73-75,80-82,92,94,100,102], SVM (linear) [41,44,45,58,60,63,87,102], SVM (radial kernel) [44,68,87], SVM (polynomial kernel) [46,87], SVM (sigmoid) [87], logistic regression (LR) [40,41,44-46,54,58-60,72,80,89,92,94,100,102], naïve Bayes [40,41,46, 52,54,58, 60,63,66, 70,73-75, 80,81,86, 91,100], random forest (RF) [40,41,45,54,58,70,73-75,82,84,86,91,92,100,102], decision tree-based classifier (DT) (eg, J48) [46,54,55,74,81,86,91], k-nearest neighbors (KNN) [54,63,66,74,84], AdaBoost [46,54,86,91], maximum entropy text classifier [79,81,94,95], sequential minimal optimization [84], multilayer perceptron [84], REPTree [88], feed-forward neural network [94], and gradient boosting [48,54,94]. One study used a supervised version of LDA called labeled LDA for text classification [87], while another utilized a supervised learning–based statistical model called the ridge regression statistical model for performing the classification task [103]. One study developed a text mining framework to evaluate data quality using a search query–based classifier and an evaluation matrix–based classifier [69]. One study used RtextTools in R (The R Foundation) for automated text classification via supervised learning [43].

One study utilized specialized software for analyzing textual content generated from online peer interactions, namely, Leximancer [90]. Few studies used packages in R for text mining, such as RWeka [43] and tm [43,68,98,99].

##### Deep Learning Techniques

Out of 64 studies, 6 (9%) used deep learning models for text classification, such as convolutional neural networks (CNNs) [41,70,73-75,100], long short-term memory (LSTM) [41,72], LSTM-CNN [41], bidirectional LSTM [41], shallow neural network [100], and reinforcement neural network–gated recurrent unit [100]. Hassanpour et al [72] optimized their deep learning model through the stochastic gradient descent optimization algorithm. One study used an ensemble deep learning model consisting of a word-level CNN and a character-level CNN [73]. One of these studies also performed image classification using image features extracted through a residual neural network [72], which is a state-of-the-art CNN architecture for computer vision tasks. Another study [87] performed image as well as video classification using a neural network called AlexNet, which is another famous deep CNN used for computer vision problems.

##### Word Embeddings: Pretraining

The following studies used pretraining with word embeddings, such as global vectors (GloVe) word vectors (ie, general domain) [41], word2vec pretrained on the Wikipedia corpus [72], and word2vec pretrained using domain-specific corpora [41,70,74,75]. One study pretrained with the image classifier model using the ImageNet data repository [72], and in another study a word-level CNN was pretrained on drug chatter word embeddings (ie, 400 dimensions) [73].

##### Empirical Distributional Semantics

Some studies applied distributional semantics to recognize meaningful relationships between terms, for instance, between messages and identified themes applying techniques such as latent semantic analysis (LSA) [64,65], random indexing (RI) [55], and the skip-gram with negative sampling (SGNS) algorithm [56] using the Semantic Vectors package. Some of these studies used pretraining on general domain corpora: RI with the Touchstone Applied Science Associates (TASA) corpus [55], the SGNS algorithm with the Wiki corpus [56], and LSA with the TASA corpus [64,65].

##### Topic Modeling

Multiple techniques were used for topic modeling, such as Quanteda software [42], LDA [49, 57,60,62, 69,77,83, 84,97-99, 101], SAS Text Miner (SAS Institute) [61,76,85,93], and correlated topic modeling, using the topicmodels package in R [86]. Out of 64 studies, 2 (3%) used the word2vec model: one to identify words similar to unigrams and bigrams per topic [47] and another for word semantic clustering [97]. One study detected topics by calculating frequency vectors to create a term-Tweet frequency table and performed chi-square tests to compare terms across the corpus [96].

Various unsupervised ML models were also utilized for identifying e-cigarette communities using k-means clustering [42] and pattern or theme recognition through a technique called the biterm topic model [78]. One study performed clustering analysis through an agglomerative hierarchical clustering technique [102] to group the temporal patterns of alcohol consumption among members of an online community.

##### Language Modeling

Out of 64 studies, 5 (8%) performed linguistic text analysis using linguistic inquiry word count (LIWC), which is used to count words in psychologically meaningful categories [45,71,83,88,89]. Linguistic analysis performed by Singh et al [45] for analyzing smoking cessation behaviors showed that interrogatives in the form of seeking information were more frequently expressed in an individual’s language if they belonged to the *contemplation* stage of behavior change; however, numbers were more frequently expressed in an individual’s language if they belonged to the *action* stage of behavior change. Another study showed that words carrying negative affect were more frequently associated with greater substance abuse [71]. In one study, LIWC was used to measure personal pronoun use within each community to understand if the individual was tweeting about one’s drinking behavior or was referencing others’ behavior [83]. One study extracted psycholinguistic features from the language used on social media platforms to train a classifier to predict recovery from alcoholism [88]. Similarly, another study showed that the negative emotions or swear words, inhibition words, and love words were significantly associated with increased risk of relapse for individuals suffering from alcohol use disorder [89].

##### Sentiment Modeling

Out of 64 studies, 20 (31%) performed sentiment analysis to gauge the positive, negative, or neutral sentiment of individuals toward health behaviors (eg, e-cigarettes, hookah, drug abuse, vaping, and JUUL) [40,41, 43, 51,59,63, 66-68,79,80,83, 85, 86,91, 93-96,103]. Some techniques used for performing sentiment analysis included SentiWordNet 3.0 [59]; the SentiWords (sentiment words) lexicon [85]; Sentiment140 [96]; maximum entropy text classifier [79,94,95]; Mathematica 10.3 (Wolfram) [93]; SVM trained on SemEval (semantic evaluation), ISEAR (International Survey on Emotion Antecedents and Reactions) emotion data sets, and on an emotion-tagged tweet corpus [51]; and various supervised ML algorithms [40,41,43,63,66,67,80,86,91]. One study calculated sentiment scores from the Liu and Hu opinion lexicon dictionary [68], one study used National Resource Council Hashtag Sentiment Lexicons to measure the positive sentiment associated with a tweet [83], and three studies used VADER (Valence Aware Dictionary and sEntiment Reasoning), which is a lexicon and rule-based sentiment analysis tool [51,80,103].

#### Model Evaluation and Metrics

To evaluate the performance of the classification models, several studies divided their data sets into training and test sets, performed n-fold cross-validations, and calculated metrics such as accuracy, precision, recall, F1 score, specificity, the Matthew correlation coefficient, and area under the receiver operating characteristics (AUROC) curve. We compiled our Results section using the F1 scores reported by various studies. If any study did not report their F1 scores, we listed the metrics they reported in their study. Most of the studies reported the F1 scores for classification tasks [40,41,43-46,48, 51, 54,55,59, 60,66-70, 72-74,80, 81,84, 87, 88,91,92, 94,95,102,103], and they ranged from 0.42 to 0.99 across various studies. Cross-validation was performed using various folds: 4-fold [59], 5-fold [67,80,82,92], 6-fold [73], and 10-fold [40, 44-46,54, 58, 60,63, 66, 68,74, 75, 81,86, 88, 91,102,103] cross-validation. Three studies reported only the accuracy values for evaluating the classifier performance [52,63,100]. One study reported only the precision of the information retrieval system [56], while two studies reported only the values obtained from AUROC curves [58,82]. One study evaluated the quality of themes identified using two approaches: supervised evaluation, by manually annotating tweets for each theme and calculating the average false-positive rate, and unsupervised evaluation, by calculating cluster purity that quantifies how coherent the theme is [78].

#### Quantitative Modeling Using Social Network Analysis

Out of 64 studies, 9 (13%) performed social network analysis [42,48,50,53,64,65,86,91,103]:

1. One study generated network graphs to visualize presence and co-occurrence of e-cigarette topics across different subreddits [42].
2. One study created network graphs to understand the reach of a campaign targeted to educate young individuals about harmful effects of smoking [48].
3. One study identified topics of e-cigarette–related conversations by creating a Twitter hashtag co-occurrence network [50].
4. One study analyzed structural differences in social networks of smokers and nonsmokers by analyzing the relationship of network metrics with smoking status of individuals [53].
5. One study performed affiliation network analysis by constructing two-mode network graphs to understand the association of the members of a smoking cessation community with different communication themes [64].
6. One study visualized topological and theme-based differences in social networks of members of an online smoking cessation community [65].
7. One study analyzed how an individual’s social network connectivity affected their alcohol use behaviors based on the topics of discussion [86].
8. One study showed that individuals who expressed negative sentiment about drinking were more centrally located within the social network compared to other members of the community [91].
9. One study quantified the peer interactions between the members of the community using social network features (eg, in-degree, out-degree, degree, reciprocity, and clustering coefficient) [103].

The tools and software programs used for performing such analysis included the Gephi platform [48,50,65]; NetworkX, a Python package (Python Software Foundation) [86]; UCINET software (Analytic Technologies) [42,64]; and the iGraph package in R [53]. One study visualized frequent word co-occurrences by creating a sociogram using NodeXL (Microsoft) [42]. Two studies did not specifically mention the tools they used for performing social network analysis [91,103]. Varying metrics were used for social network analysis, such as degree centrality [42,64], modularity [48,65], and in-degree and out-degree centralities [86,91]. One study used multiple metrics for analyzing social network structures, such as vertices, edges, density, isolates, diameter, communities, betweenness centrality, closeness centrality, transitivity, clusters, and modularity [53]. Table 3 [40-46,48-55,57-89,91-103] highlights the summary of methodological functions used across various studies and also lists the specific tools used for performing those functions.

**Table 3:** Summary of methods and related tools used by various studies.

Methods	Tools, platforms, and programs
Linguistic analysis	Linguistic inquiry word count [45,71,83,88,89]
Sentiment analysis	SentiWordNet 3.0 [59] SentiWords (sentiment words) lexicon [85] Sentiment140 [96] Maximum entropy text classifier [79,94,95] Mathematica 10.3 [93] Various supervised machine learning algorithms [40,41,43,51,63,66,67,80,86,91] Liu and Hu opinion lexicon dictionary [68] VADER (Valence Aware Dictionary and sEntiment Reasoning) [51,80,103] National Resource Council Hashtag Sentiment Lexicon [83]
Supervised classification	Support vector machine [41,44-46,51,54,58,60,63,66-68,70,73-75,80-82,87,92,94,100,102] Logistic regression [40,41,44-46,54,58-60,72,80,89,92,94,100,102] Naïve Bayes [40,41,46,52,54,58,60,63,66,70,73-75,80,81,86,91,100] Random forest [40,41,45,54,58,70,73-75,82,84,86,91,92,100,102] Decision tree-based classifier [46,54,55,74,81,86,91] k-nearest neighbors [54,63,66,74,84] AdaBoost [46,54,86,91] Sequential minimal optimization [84] Maximum entropy text classifier [79,81,94,95] Multilayer perceptron [84] REPTree [88] Feed-forward neural network [94] Gradient boosting [48,54,94] Convolutional neural networks (CNNs) [41,70,72-75,87,100] Long short-term memory (LSTM) [41,72] LSTM-CNN [41] Bidirectional LSTM [41] Shallow neural network for text classification [100] Reinforcement neural network–gated recurrent unit [100]
Topic modeling	Quanteda software [42] Latent Dirichlet allocation [49,57,60,62,69,77,83,84,97-99,101] SAS Text Miner [61,76,85,93] Correlated topic modeling [86]
Community identification and theme or pattern recognition	k-means clustering [42] Biterm topic model [78] Agglomerative hierarchical clustering technique [102]
Social network analysis	Gephi platform [48,50,65] NetworkX (Python package) [86] UCINET software [42,64] iGraph package in R [53] NodeXL [42]

## Discussion

### Principal Findings

The purpose of this review was to investigate the current state of computational and quantitative techniques available for analyzing risky health behaviors, beliefs, and attitudes using online peer interactions from social media platforms. From the initial set of studies retrieved and snowballing techniques, 64 studies that met our inclusion criteria were included in this review, out of which 75% (48/64) [40-57,68-79, 82-94,97, 98, 100-102] were published in 2017 onward. This suggests that there is a growing trend in utilizing computational approaches to characterize risky health behaviors by analyzing conversational data generated from online peer interactions.

Several platforms were used as the source of data for analyzing risky health behaviors, with the most popular being open social media platforms, since 80% (51/64) of the studies utilized them as compared to intentionally designed health-related social media platforms. In terms of data collection, our results showed that Twitter was a popular source of social media data, as it provides three easy ways to access the data: Twitter Search API, Twitter Streaming API, and Twitter Firehose [104]. Some studies utilized platforms (eg, Facebook, Instagram, and Reddit) that also provide access to data through their APIs [105-107] but were not as widely used as compared to Twitter. A few studies utilized intentionally designed health-related social media platforms, such as QuitNet, Cancer Survivors Network, patient.info/forums, BecomeAnEx.org, Hello Sunday Morning blog, and the A-CHESS online discussion forum, but they did not provide any information about their data collection techniques. In terms of data types, this review included studies that primarily focused on analyzing textual data generated from online peer interactions. Thus, we excluded two studies during the full-text screening that focused on analyzing risky health behaviors through image analysis only [108,109].

Sentiments toward smoking-related products (eg, cigars, e-cigarettes, hookah, vaping, and JUUL) and identification of various themes related to the discussion of such products were widely studied using online social media platforms. Prescription drug abuse, opioid misuse, and binge drinking–related behaviors were another set of widely analyzed risky health behaviors using online social media platforms. This highlights the potential of using such platforms for the dissemination of behavioral change interventions targeting uncharted and evolving domains (eg, e-cigarettes) as well as well-charted domains (eg, alcohol use). In addition to addictive behaviors, uptake behaviors were analyzed, such as the association of physical activity patterns, sentiments, and types of behaviors (eg, running, walking, and jogging) with different geographical locations (eg, in Canada) and population demographics (eg, genders). Social media platforms were used for identifying the themes related to weight loss and obesity-related behaviors. None of the studies focused on analyzing unprotected sex–related behaviors, an important public health focus and priority, which can likely be an interesting avenue for future research. However, given the stigma, privacy concerns, and the opaque nature of the domain, access to such data sets might be limited.

The LIWC tool was widely used for linguistic feature extraction, as it is an easily accessible tool that extracts features like style words, emotional words, and parts of speech from the texts [110]. Language modeling performed using LIWC showed how the usage of language among members can be used to predict their relapse or behavior transition patterns. For topic modeling, LDA was the most commonly used tool; it analyzes latent topics based on word distribution and then assigns a distribution of topics to each document [111]. The topics discussed varied from one risky health behavior to another but mostly highlighted the attitudes and behavior patterns of individuals engaging in such behaviors. Few examples include highlighting the controversial topics related to e-cigarette and marijuana use (eg, legalization, prohibition, etc) [101], identifying topics related to the normative or cultural context surrounding e-cigarette use and alcoholic preferences [60,83], and understanding how the social environment of individuals affects their behaviors toward weight loss [98].

A wide range of supervised ML algorithms were used for the content and sentiment analysis of the data generated from online peer interactions. Most of the studies utilized traditional ML models (eg, SVM, LR, RF, DT, and KNN) for text classification purposes. Only a few studies [41,70,72-75,87,100] utilized deep learning models (eg, CNNs and LSTMs) for text as well as image and video classification tasks. In terms of performance evaluation, the following results were observed:

1. In 4 out of 64 (6%) studies [41,72-74], the performance of deep learning models on classification tasks was better compared to the traditional ML classifiers (eg, the deep learning model had an AUROC curve of 0.65 as compared to the baseline LR model, which had an AUROC curve of 0.54 [72]).
2. In 1 study out of 64 (2%) [75], the deep learning model marginally outperformed the traditional ML classifier: RF (accuracy 70.1%) and deep CNN (accuracy 70.4%).
3. In another 2 studies out of 64 (3%) [70,100], the performance of deep learning models on classification tasks was lower compared to the traditional ML classifiers (eg, RF [accuracy 93.4%] performed better than CNN [accuracy 60.1%] [100]).

The majority of the studies included in this review focused only on textual data analysis of online peer interactions, while only one study performed additional analysis using image data [72], and only one performed textual, image, and video data analysis [87]. Few studies [41,55,56,64,65,70,72-75] created word vectors using pretrained word embeddings (eg, GloVe, word2vec, drug chatter word embeddings, LSA, RI, and SGNS). These were trained using different types of corpora (eg, the Wikipedia corpus [56,72], the TASA corpus [55,64,65], or a domain-specific corpus [41,70,72,74,75]). The performance of classifiers using pretrained word embeddings ranged from 0.99 to 0.55 in terms of F1 scores.

Some of the studies included in this review also performed network analysis [42,48,50,53,64,65,86,91,103]. The Gephi platform [112] and UCINET software [113] were widely used tools for analyzing online social ties. One study characterized the role of content-specific social influence patterns underlying peer-to-peer communication using affiliation exposure models and the two-mode version of the network autocorrelation model [64]. One study analyzed the social network structure of smokers and compared it with the network structure of nonsmokers to understand the factors related to the social influence that might affect addictive tobacco-related behaviors [53]. Such network analysis can help us understand the context of communication, which can eventually guide the development of tangible technology features by health researchers and technology developers [114,115].

One study [85] analyzed online peer interactions based on a communication model called the dynamic transactional model [116], which is suitable for modeling two-way communication between individuals. Very few studies [42,45,55,64,65,97] linked theoretical constructs that define behavior change in analyzing content generated from social media platforms, such as social cognitive theory [117], the transtheoretical model of change [118], the health belief model [119], and the taxonomy of behavior change techniques [120]. The online peer interactions should be analyzed using theoretical frameworks that can lead to the development of empirically grounded digital health interventions for promoting health and positive behavior changes [121,122]. Theory-driven large-scale analysis of social media data sets will yield insights into the specific processes of behavior change that manifest in peer interactions. The analysis of these data sets in conjunction with theoretical constructs can aid in enhancing our knowledge of how social influence plays a major role in diffusing health information and modifying individual health behaviors. This can have implications for the development of high-yield interventions for individuals and populations based on their risky health behavior, thereby enabling individuals to make positive lifestyle changes and improving their quality of life.

It is also important to understand that online social media platforms can be used for disseminating health-related misinformation as well [123]. The COVID-19 pandemic has provided us with abundant evidence that highlights the urgency to address public concerns related to misinformation that is plaguing social media, which can negatively impact health-related behaviors of individuals [124,125]. Also, the ground truth of aggregated trends extracted from information disseminated through these platforms is reflective of community perceptions only to a certain extent because of the large amount of content push by automated bots [126]. Studies have shown how misinformation also impacts risky health behaviors (eg, misleading marketing claims about e-cigarettes [127] and alcohol use [128]). Future work should focus on leveraging the techniques described in this review for analysis of misinformation diffused throughout online social media platforms to enhance the utility and positive impact of these platforms.

### Limitations

Our review is not without limitations. Firstly, we included studies related to risky health behaviors alone; however, studies focusing on other public health domains (eg, epidemiology [129] and surveillance [130]) or studies focusing on chronic health conditions (eg, diabetes [131,132] and cancer [133]), as well as clinical and health outcomes [134,135], can provide us with a comprehensive understanding of how data generated from social media platforms are analyzed for various public health applications by leveraging computational modeling and high-throughput analytics. The domain of infodemiology and infoveillance is quite broad and includes various other aspects of risky health behaviors that were not included in this review (eg, mining consumer opinions toward online marketing of e-cigarettes [136,137], or understanding their reactions toward media coverage [138,139] or policy regulations [140,141] concerning such products). Secondly, we only focused on studies that primarily performed textual data analysis. Even though we did include studies that reported image or video data analysis along with textual data analysis [72,87], we did not include studies that solely described image or video data analysis [108,109]. These studies can provide useful insights into ML trade-offs and computational scalability as related to varying data density, heterogeneity, and inferential granularity.

Finally, given the constraints of our search strategy, we might have missed some studies from the infodemiology and infoveillance domain; for example, an initial exploration of the literature search in this domain [142] had resulted in a total of 397 studies, out of which 23 studies were relevant for inclusion in this review. Of these, 15 studies were captured by our search strategy and included in the review [40,41,43,50,51,54,61-63,66,68-70,80,95], and an additional one was included as part of the snowballing efforts [47]. However, the remaining seven were not identified by our search strategy [143-149]. Broad methodological descriptions or excessively granular terminology use capturing ML methods in metadata, titles, abstracts, and keywords are noted in these studies. For consistency and to limit bias with studies in other journals, we have not included these studies in the review. Future researchers conducting similar reviews should ensure the inclusion of terms that capture the interdisciplinary nature of studies (eg, infodemiology), analytical functions (eg, text classification, content analysis, and topic modeling), and analytical techniques (eg, LDA) for the exhaustive representation of related works that leverage SMaaRT for risky behavior modeling and analysis.

### Conclusions

Our review shows that online discourse related to risky health behaviors on social media platforms can span multiple topics that include nicotine dependence, alcohol use, drug or substance abuse, physical activity patterns, and obesity-related behaviors. This results in the generation of large amounts of digitally archived data, which can provide a deeper understanding of the organic manifestation and natural evolution of health-related behavior change processes.

Our review highlights the characteristics of social media platforms (eg, general-purpose vs health-focused platforms and ease of data access for secondary analysis), the robustness of methods used for analyzing peer interactions within these platforms, and an overview of a wide variety of text mining and network modeling tools available to conduct analyses of social media data sets at scale. Our review allows us to consolidate the methodological underpinnings and enhance our understanding of how social media can be leveraged for nuanced behavioral modeling and representation. This can ultimately inform and lead to the formulation of persuasive health communication and effective behavior modification technologies targeting inter- and intrapersonal psychosocial processes distributed at the individual and population levels. It is also important to understand the merits and shortfalls of existing computational studies to assess the generalizability and strength of the downstream predictive models and data-driven interventions resulting from such large-scale analyses.

## Appendix

Multimedia Appendix 1 A detailed summary of the studies included in the review.

## Abbreviations

A-CHESS: Addiction–Comprehensive Health Enhancement Support System
API: application programming interface
AUROC: area under the receiver operating characteristics
CNN: convolutional neural network
DT: decision tree
GloVe: global vectors
ISEAR: International Survey on Emotion Antecedents and Reactions
KNN: k-nearest neighbors
LDA: latent Dirichlet allocation
LIWC: linguistic inquiry word count
LR: logistic regression
LSA: latent semantic analysis
LSTM: long short-term memory
MeSH: Medical Subject Headings
ML: machine learning
PRISMA: Preferred Reporting Items for Systematic Reviews and Meta-Analyses
RF: random forest
RI: random indexing
SemEval: semantic evaluation
SentiWords: sentiment words
SGNS: skip-gram with negative sampling
SHAP: SHapley Additive exPlanations
SMaaRT: social media as a research tool
SVM: support vector machine
TASA: Touchstone Applied Science Associates
VADER: Valence Aware Dictionary for sEntiment Reasoning
