# Supplementary material for: Social Media as a Research Tool (SMaaRT) for Risky Behavior Analytics: Methodological Review
Source: JMIR Public Health Surveill. 2020 Nov 30;6(4):e21660. doi: 10.2196/21660 (PMC7735906; doi:10.2196/21660)
Supplement: Multimedia Appendix 1 [file publichealth_v6i4e21660_app1.pdf]

Multimedia Appendix 1. A detailed summary of the studies included in the review.

First Author, Year, Reference #	Risky Health Behaviors	Social Media Platforms	Number of Posts	Study Focus	Key Methodological Functions / Tools	Evaluation Metrics Used	Key Findings
Benson, 2020 [40]	Nicotine Use	Twitter	Total - 11,556 tweets (July 2018 – August 2019)<br><br>Manually annotated - 4000	To understand the thematic interests and health perceptions (sentiments) of underage individuals towards JUUL use	Feature Extraction – n-grams (unigram, bigram, trigram)<br><br>Automated classification of tweets into three categories – underage JUUL use, positive and negative sentiment<br><br>Classifiers – LR, RF, Bernoulli NB<br><br>Feature selection – SelectKBest<br><br>Hyperparameter optimization – GridSearchCV (10-fold cross validation)	ACC, PR, RC, F1 score	The most prevalent category of JUUL-related usage corresponded to first person usage or experience from the annotated tweets (56.85%). Very few annotated tweets (1.43%) mentioned using JUUL as a method of smoking cessation. Some tweets (6.85%) also mentioned the health benefits or detriments of using JUUL. Majority of the annotated tweets (44.92%) contained neutral sentiment towards JUUL, overall the sentiment was more positive (33.37%) than negative (18.59%) towards JUUL usage.<br><br>For all three classification tasks, RF outperformed other classifiers – Underage JUUL use (F1 score – 0.99), Positive sentiment (F1 score

							– 0.82), Negative sentiment (F1 score – 0.91). Overall model F1 score was 0.91.
Visweswaran, 2020 [41]	Nicotine Use	Twitter	Two datasets – 1) Total - 810, 600 tweets, out of which 4000 were manually annotated for traditional classification (August 2018 – October 2018) 2) 1,899,851 tweets to derive word vectors for deep learning (January 2018 – July 2018)	To evaluate the potential of various ML classifiers for automated identification of vaping-related tweets	The tweets were annotated for three levels – at the first level whether the tweet is relevant or not, only the relevant tweets are further annotated as commercial or not, only non-commercial tweets are further annotated as having pro-vape sentiment or not<br><br>Traditional Classification:<br>Text pre-processing – removal of textual placeholders, removal of punctuation, normalization, etc.<br>Feature Extraction – Count vectors, TF-IDF Vectors<br>Classifiers – LR, RF, SVM (linear), NB<br><br>Deep Learning Classification:	Evaluation of classifiers - AUROC, PR, RC, F1 score<br><br>Evaluation of relevant features for each classifier – SHAP (Shapley Additive explanation)	Annotated dataset consisted of 75.28% vaping related tweets, out of which 72.24% were non-commercial in nature, out of which 61.39% had a pro-vape sentiment.<br><br>Relevance Classification – LSTM-CNN with GloVe word vectors gave the highest performance (F1 score – 0.98).<br><br>Commercial Classification - LSTM-CNN and BiLSTM with GloVe word vectors gave the highest performance (F1 score – 0.99, 0.98 respectively).<br><br>Sentiment Classification - LSTM-CNN and BiLSTM with GloVe word vectors gave the highest performance (F1 score – 0.84, 0.88 respectively).<br><br>The analysis of 10 top-ranked features showed that

					Text pre-processing for deep learning was done using two methods – fixed pre-processing pipeline OR no pre-processing.<br>Word Vectors – 200-dimension GloVe (general domain) and 300-dimension word2vec vaping related word vectors (using the second dataset)<br>Classifiers – CNN, LSTM, LSTM-CNN, BiLSTM (bidirectional LSTM)		similar features appear across the classifiers.
Barker, 2019 [42]	Nicotine Use	Reddit	Total - 79,783 submissions / 5018 unique subreddits (March 2017 – February 2018)<br><br>For identifying e-cigarette communitie	Identify commonly discussed topics about e-cigarettes and vaping and analyze the distribution of these topics across different communities across Reddit	Identifying submission topics – Quanteda software package was used to create and e-cigarette topics dictionary using all the submissions<br><br>Identifying e-cigarette communities – k-means clustering<br><br>SNA – UCINET	Identifying submission topics and communiti es - Inter-rater reliability (α) was calculated between human and machine classificatio n	Seven e-cigarette-related topics were identified from all the submissions – advice (15%), build you own (17%), buying/selling (19%), drugs (7%), e-juice (17%), health/safety (5%), and tobacco (7%).<br><br>Six discrete communities were identified from 2989 subreddits – general e-cigarettes (9%), vendor/sales (8%), drugs (8%), research/news (6%),

			s - 2989 subreddits		Quadratic assignment procedure (QAP) correlation analysis - to analyze the extent to which communities were similar in their depictions of various e-cigarette topics<br><br>Word co-occurrence visualization – Sociogram using NodeXL	SNA – Degree centrality<br><br>QAP analysis - Statistical significance level of .05	advice (5%), and other (64%).<br><br>Based on full network data, the most central topic was related to buying or selling on e-cigarettes (10,346) followed by e-juice (10,250), advice (8484), and build you own (8357).<br><br>The results of QAP analysis showed that the general e-cigarette community shared significant similarities in their depictions of e-cigarettes and with the vendor (r = .83, p < .01), advice (r = .63, p < .01), and other (r = .85, p < .001) communities.
Chu, 2019 [43]	Nicotine Use	Twitter	Total - 561,960 tweets (January 2016 – June 2016)<br><br>Manually annotated – 5000	Analyze the sentiment towards HTS on Twitter and identify individuals who have mixed opinions about these products to help public health officials with efficient dissemination of tailored HTS-related educational messages	Linguistic feature extraction (language-based covariates) - RWeka and tm packages<br><br>Automatic Text Classification via Supervised Learning – RTextTools (Sentiment analysis to identify	PR, RC, F1 score	The positive sentiment tweets (67%) were identified with F1 score of 0.86 and negative sentiment tweets (33%) were identified with F1 score of 0.67. A total of 4.41% members were identified as having both positive and negative sentiment towards HTS after the results of ML classification. After

					positive and negative sentiment tweets)		qualitative analysis, 37 individuals were confirmed to have ambivalent views.
Czaplicki, 2019 [44]	Nicotine Use	Instagram	Total – 14838 posts (March 2018 – May 2018)<br><br>Manual Annotation – 1500 for relevance classification, 1104 for promotional content and nicotine and addiction-related content	Analyzing the amount and characteristics of JUUL-related posts (a popular vaping device). Content analysis was done for the following categories: promotion, nicotine and addiction, and youth-related appeals and lifestyle, and social norms and acceptability-related content. Both visual and textual/language content of the posts were analyzed in this study.	JUUL Relevance Classification<br>Features – unigrams, number of hashtags, number of hashtags containing strings such as “juul” or “vap”<br>Linear SVM with bagging was trained (10-fold cross validation)<br><br>Content Classification<br>Features – unigrams, bigrams, trigrams, usernames, hashtags, language-based filter for lifestyle or social norms and acceptability-related content<br><br>LR with L1 regularization (for promotional content, nicotine and addiction-related content)<br>SVM with radial kernel (for youth-related content)	ACC, PR, RC, F1 score	Relevance classifier achieved F1 score of 0.92, and 37% of total posts were classified as being relevant to JUUL. Promotional content classifier achieved F1 score of 0.84, and 34% of total posts were classified as promotional. Nicotine and addiction-related content classifier achieved F1 score 0f 0.75, and 11% of total posts were classified as nicotine and addiction-related. Youth-related content classifier achieved F1 score 0f 0.83, and 55.4% of total posts were classified as youth-related. The lifestyle-related content filter achieved F1 score of 0.85, and 57% of the posts mentioned lifestyle-related content. There are also posts from young individuals regarding nicotine and addiction that are tied into youth culture and lifestyle appeals using hashtags, memes, friend-tagging for

					Language-based filter (for lifestyle or social norms and acceptability-related Messages)<br>(10-fold cross validation)		promotion, tag lines. 71.9% of promotional posts contained lifestyle-related content.
Singh, 2019 [45]	Nicotine Use	QuitNet	Total - 111,004 journal text entries (1999 - 2015)<br><br>Manually annotated - 500	Identify stages of behavior change according to the Transtheoretical Model (TTM) of change using recorded journal entries of members of an online smoking cessation community	Feature Extraction – word2vec<br><br>Classifiers – LR, SVM (linear), and RF<br>(10-fold cross validation)<br><br>Linguistic Analysis - LIWC	RC, PR, F1 score	RF classifier gave the highest performance with F1 score of 0.90 as compared to other classifiers. Linguistic analysis showed that interrogatives were more prevalent in the ‘Contemplation’ stage of behavior change (e.g. seeking information), while numbers were highly used in the ‘Action’ stage of behavior change which may be due to quantities expressed to show progress in terms of number of cigarettes not smoked or number of abstinent days. Sense of achievement and work- induced stress (obstacles to quitting) were emphasized in the ‘Action’ stage.
Wang, 2019 [46]	Nicotine Use	BecomeAnEX.com	Total – 38,156 blog posts and 361,886 blog	Identify an individual’s smoking status using member-generated content from an online smoking cessation	Feature Extraction - 5 different feature sets were extracted for ML: Standard text features (unigrams) of focal	F1 score and AUROC	The best overall performance across all the models and algorithms for identifying smoking status was achieved by model with feature sets 3,

			comments (January 2012 – May 2015)<br><br>Manually annotated - 2120	community with the goal of tailoring smoking cessation intervention as per an individual's needs.	post content (Feature set 1)<br>Doc2Vec features (200 dimensional) from focal post content (Feature set 2)<br>Domain-specific features from focal post content (Feature set 3)<br>Author-based features of focal posts (Feature set 4)<br>Thread-based features of a focal post's thread (Feature set 5)<br><br>Classifiers - NB, LR, J48 decision tree, SVM (with polynomial kernel), and AdaBoost with two weak learners (DecisionStump and J48)<br>(10-fold cross validation)		4, and 5 and with AdaBoost algorithm using DecisionStump as the weak learner. This combination yielded an F1 score of 0.837. These feature sets helped to improve the performance of the classifier by 9.7% as compared to using only textual features of post's content. This classifier was also able to identify abstinence status of 60% of members – those who had authored a blog or blog comment with at least one post indicating a period of abstinence.
Allem, 2018 [47]	Nicotine Use	Twitter	Total - 176,706 tweets (April 2017 – March 2018)	Characterize users' experiences with hookah and understand the social and environmental	Topic identification –<br><br>Tweets were initially analyzed through unigram and bigrams frequencies		Based on word frequencies the following topics were identified – person tagging, buying or selling, appeal or abuse liability, hookah use behavior, promotional or

				context in which hookah is being used	Used word clouds for visualizing the common topics<br><br>Language modeling / word embeddings – word2vec to identify words similar to unigrams and bigrams per topic in word cloud stage<br><br>Rule-based classification script to check for existence of specified set of n-grams representing a topic		social events, polysubstance use, flavors and dislike of hookah. These topics constituted 65.45% of all the tweets. The most prevalent topic was person tagging followed by a promotional or social events, appeal or abuse liability and hookah use behavior. Cannabis use was another common topic, raising major public health concerns as little cigars may be at the intersection of nicotine and cannabis use and thus lead to nicotine addiction.
Chew, 2018 [48]	Nicotine Use	Twitter	9500 accounts following @knowtherealcost, 200 tweets from each account were collected for age prediction	Perform social network analysis to understand the reach of The Real Cost Twitter campaign (@knowtherealcost) by identifying core network communities and examine the age distribution for the members of those communities to predict if those members are youth vs. young adult's	Graphing Network – created 1.5-degree ego network including only follower accounts that were connected to at least one other follower account using network analysis software Gephi- (ForceAtlas2 layout algorithm). For community detection Louvain method was used	Modularity for community detection<br><br>Age prediction model performance – PR, RC, F1 score	The four key communities among the @knowtherealcost Twitter followers were music, Tony Hawk/skating, public health, and ABC's The Fosters. The age prediction model had precision, recall and F1 score of 74%. The greatest proportion of youth were members of the ABC Foster's Show, followed by the skating and music communities.

				vs adults. This campaign was targeted to educate young individuals about harmful effects of smoking.	Age Prediction Model – Gradient Boosting Tree Classifier		
Zhou, 2018 [49]	Nicotine Use	Facebook	Total – 765,321 posts, 2,737,840 comments (upto April 2015)	Understand how diffusion of flavor-related information influence behaviors towards e-cigarettes. Characterize the influence of flavors on information diffusion of e-cigarette related posts.	Topic Modeling – LDA<br><br>Statistical Analysis – Regression Models<br><br>Dependent Variable - Frequency of being shared by Facebook users for each post (information propagation)<br><br>Independent Variables 11 binary variables for flavor categories<br><br>Control Variables – Binary dummy variable promotion, Count of posts in a Facebook page, Average Share, Different topics, URL mention, Hashtag		Most frequent words for each of the three topics were as follows:<br>Topic 1 (details about e-cigarette) – new, now, flavor, stock, available, etc.<br>Topic 2 (methods of e-cigarette consumption) – vape, vapor, hookah, juice, etc.<br>Topic 3 (related discussions) – tobacco, smoking, help, vaping, etc.<br><br>The hurdle negative binomial regression model as the base model to characterize the relationship between the independent and dependent variables. Flavors such as sweet, dessert & bakery, fruits, herbs & spices, and tobacco has negative influence on propagation of e-cigarette related posts. No flavor had a positive

							significance on information propagation.
Allem, 2017 [50]	Nicotine Use	Twitter	Total – 2,190,672 tweets (December 2016 – April 2017)	Identify themes of discussion related to e-cigarettes as well as identify locations where e-cigarettes are mostly discussed to be able to identify priority areas for public health educational campaigns	Distinguish tweets of human users from social bots - BotOrNot algorithm<br><br>Discussion Themes Identification through SNA - Hashtag co-occurrence network using Gephi with Fruchterman Reingold force-directed layout. For community detection Louvain method was used<br><br>Statistical Analysis - Odds ratios were computed to detect if hashtags varied among social bots and human users using the Fisher exact test<br><br>Location Identification – latitude and longitude coordinates from metadata, followed by creation of heat map showing		Three main clusters were identified from the bot-free hashtags corpus: Cluster 1 was related to behaviors (e.g. #vaping), Cluster 2 was related to vaping products, vaping identity (e.g. #vapelife), and vaping community (e.g. #vapenation), and Cluster 3 was related to tobacco use and polysubstance use.<br><br>Odds ratio analysis showed that bots were more likely to post hashtags that referenced smoking cessation and new e-cigarette devices compared to human users.<br><br>The heat maps showed that a large number of tweets were from Mid-Atlantic and Southwest geographical regions and make them priority areas for educational campaigns.

					frequency of tweets by location		
Allem, 2017 [51]	Nicotine Use	Twitter	Total - 591, 792 tweets (March 2015 – December 2016)	Sentiment analysis of hookah-related posts on Twitter to understand the attitudes of users towards Hookah	Sentiment Analysis – 2 different methods were used:<br><br>Baseline model was based on rule-based reasoning (VADER)<br><br>Automated inference logic model such as SVM (trained on SemEval, ISEAR emotion datasets and on emotion-tagged tweet corpus)	Baseline and automated model – F1 score	The rule-based reasoning sentiment analysis achieved an F1 score of 0.96. The SVM model achieved an F1 score of 0.90. Positive, negative and neutral opinion tweets were identified, the majority being positive (59.5%). Positive tweets were further classified into highly positive emotions (active, happy, etc.) and passive positive emotions (calm, relaxed, etc.) Negative tweets were classified into subdued negative emotions (sad, bored, etc.) and highly negative emotions (stressed, nervous, etc.).
Dai, 2017 [52]	Nicotine Use	Twitter	Total – 757,167 tweets (July 2015 – October 2015)<br><br>Manually annotated – 2000	Evaluate the general public's attitudes/opinions towards e-cigarettes. To compare public opinions by geographical regions and identify potential socioeconomic factors that impact public perceptions about e-cigarette use	Automated text classification - the tweets were classified into 5 polarities - against, support, neutral, commercial, irrelevant<br><br>Classifier – multilabel NB model	Classifier – ACC<br><br>Statistical analysis - p value <0.05 was considered statistically significant	The classifier achieved an accuracy of 93.6% on the training data. The results showed that general public had mixed opinions about e-cigarettes. Overall, amongst organic tweets, there were about 17.7% against tweets, 10.8% support tweets and 19.4% neutral tweets. The organic—against tweets mostly focused on delivering

					GIS Analysis - The metadata was used to evaluate geographical locations of e-cigarette–related tweets<br><br>Six socioeconomic factors from 2014 American Community Survey (ACS) data were used to investigate the potential socioeconomic impact on public perceptions about e-cigarette use. Pearson correlation between prevalence and opinion polarities was calculated		strong educational information about the risks of e-cigarette use and encouraged public, especially youth, to stop vaping. However, the organic—against tweets were outnumbered by commercial tweets and organic—support tweets combined. In terms of spatial analysis, Canada (61.6%) had the highest number of commercial tweets whereas Malaysia (27.8%) had the lowest number of commercial tweets. Higher prevalence of organic tweets was associated with states with higher education rates (r=0.60, p<0.0001), higher percentage of black and African-American population (r=0.34, p=0.01), and higher median household income (r=0.33, p=0.02).
Fu, 2017 [53]	Nicotine Use	Facebook	Social networks were created using Facebook profiles of	Identifying the structural characteristics of online social networks of smokers and non-smokers and understanding	SNA – iGraph 1.0.0 package in R<br><br>4 kinds of networks were constructed – friendship, family,	Network metrics studied: vertices, edges, density, isolates,	The results of the study showed that the four networks had different characteristics. For friendship networks, the networks of smokers had lower betweenness, lower,

			9042 smokers and 2087 non-smokers	whether the structural differences in the networks of individuals are related to their smoking status.	photo and group networks<br><br>Multicollinearity – vif function in R package<br><br>Analyze the relationship of network metrics and smoking status – LR (using R)	diameter, communities, betweenness centrality, closeness centrality, transitivity, clusters, and modularity	transitivity, and bigger diameter as compared to non-smokers. For family networks, the odds of smoking increase if the social network contains more vertices, less transitivity, and fewer isolates. No such metrics were predictive in case of photo networks and were thus similar for both smokers and non-smokers. In case of group networks, the smoker networks were smaller in diameter as compared to non-smokers.
Kim, 2017 [54]	Nicotine Use	Twitter	Total – 11.5 million tweets (November 2014 – October 2016)<br><br>4897 users were manually classified according to the user type definitions	Classify users who tweet about e-cigarette–related topics into five different categories: individuals, vaper enthusiasts, informed agencies, marketers, and spammers based on their metadata and tweeting behaviors	Feature Extraction – User Metadata features (followers count, friends count, retweet count, etc.)<br>Derived behavior features (unique keyword count in original tweets, unique keyword count in hashtags in original tweets, language count in original tweets, etc.)<br><br>Classifiers – Gradient Boosting Regression Tree, SVM, LR, RF,	F1 score	Gradient boosting classifier achieved the highest F1 score (82.5%). The model using both metadata and behavior features was able to predict members with following F1 scores - individuals (91.1%), informed agencies (84.4%), marketers (81.2%), spammers (79.5%), and vaper enthusiasts (47.1%). In terms of the top features for each member type: Individuals like more tweets, informed agencies have more followers, marketers use more e-cigarette words in

					KNN, AdaBoost, NB, DT, Dummy Classifier (10-fold cross validation)		their original tweets, vaper enthusiasts retweet e-cigarette content more and more frequent tweeting behavior is indicative of spammers.
Sridharan, 2017 [55]	Nicotine Use	QuitNet	Two datasets used –<br><br>1) 16,492 forum messages (March 2007 – April 2007)<br>2) 65,910 forum messages (January 2014 – December 2014)<br><br>Manually annotated - 1000	Characterize temporal trends in user communication by mapping the content generated through online peer interactions to theoretical behavior change constructs	Word Vector representations – utilization of TASA corpus to generate QuitNet message vector representations, variant of RI was used to recognize meaningful relationships between terms (Semantic Vectors package)<br><br>Classifier - J48 tree (Weka)<br><br>Temporal Modeling for relapsed members (status change from ex-smoker to active smoker) using annotated messages from automated analysis	PR, RC, F1 score	J48 classifier achieved F1 score of 0.77. The most commonly used themes were 'feedback and monitoring' and 'comparison of outcomes' in the years 2007 and 2014. The messages belonging to the theme 'comparison of outcomes' had increased by 22% from 2007 to 2014 which indicates that members of the community are more aware of the outcomes of behavior change associated with smoking cessation. The theme that had a dip in percentage of messages was "comparison of behavior" by 9.31%. There was a reduction in frequency of messages posted before and after relapse across all themes for the year 2007. Similar pattern was observed for the year 2014. The drop was 75% for themes - "goals

							and planning" and "feedback and monitoring" and 66.6% for themes - 'social support', 'comparison of behavior' and 'comparison of outcomes in the year 2007. Similar patterns were observed for the year 2014.
Sridharan, 2017 [56]	Nicotine Use	QuitNet	Total - 2,467,550 messages (2000 - 2015)	Analyze peer interactions to understand psychosociobehavioral factors affecting tobacco use amongst QuitNet members based on two topics - pharmacotherapy and alternative nicotine intake mode. Also, visualize the changes in those discussion topics over time from 2000 – 2015	Word Embeddings - Skipgram with Negative Sampling (SGNS) algorithm using Sematic Vectors package, pretrained on Wikipedia corpus<br><br>Information Retrieval (IR) System - Topic specific representational terms were used to obtain nearest neighboring messages<br><br>To observe trends in topics top 10 nearest neighbors of each representational term was manually analyzed. Semantic Vectors package was utilized to rank retrieved messages in	IR System – PR<br><br>Trends in topics – Z-score	The average precision for all representational terms for "modes of nicotine intake" was 0.64 and for "pharmacotherapy" was 0.74. The representational terms for "modes of nicotine intake" were e-cigarettes, snus, chew, cigars, hookah, kreteks, pipe. The representational terms for "pharmacotherapy" topic included NRT, injection, gums, patches, lozenges. The "mode of nicotine intake" topic swings back and forth from the years 2000 – 2009 and after that, there were more mentions about alternative forms of intake. For topic "pharmacotherapy", there was negative trend associated with this topic in

					order of their semantic similarity to the search terms		2000 but its mentions rise up from 2010-2015.
Westmaas, 2017 [57]	Nicotine Use	Cancer Survivors Network (CSN)	Total - 468,000 posts, 48,779 threads (2000 - 2013)<br><br>After filtering, 3998 unique posts that contained smoking-related terms remained for further analysis	Understand the concerns of cancer survivors towards smoking or quitting and identify the motivators and barriers to quitting by analyzing their online interactions with the ultimate goal to inform the cessation interventions	Topic Modeling – LDA		70 topics identified from 3998 posts (0.85% of all posts) were broadly categorized as follows - (1) experiences with cancer treatment and side effects (32.4% of the topics), (2) health behaviors (16.2%), (3) emotional expressions (11.9%), (4) social support (10.3%), (5) biological functioning (9.8%), (6) causes of cancer (5.6%), and (7) experiences with healthcare (4.2%). There was a low frequency of smoking/cessation-related posts out of which most frequent were quit smoking methods (5.4%), environment for quitters (2.9%) and smoking as a risk factor for one's diagnosis (1.9%).
Aphinyanaphongs, 2016 [58]	Nicotine Use	Twitter	Total – 13,146 tweets (January 2010 –	Automate the identification of tweets that indicate e-cigarette use and tweets that indicate e-	Feature Extraction – unigrams, bigrams<br><br>Classifiers - NB, SVM (linear), Bayesian LR, and RF	AUROC	LR (0.90), SVM (linear) (0.87) and RF (0.89) performed with high area under the receiver operating curve for identification of tweets that indicate e-

			January 2015)<br><br>Manually annotated - 1000	cigarette use for smoking cessation	(10-Fold Cross Validation)<br><br>Keyword comparisons used to compare ML models to simple keyword-based approach for identifying tweets		cigarette use. RF (0.94) performed with high area under the receiver operating curve for identification of tweets that indicate e-cigarette use for smoking cessation. Keyword based searches were inferior to the ML methods.
Kavuluru, 2016 [59]	Nicotine Use	Twitter	Two datasets used –<br><br>3) 224,000 tweets (September 2013 – December 2013)<br>4) 1 million tweets (March 2015)<br><br>Manually annotated - 1000	Identify the proponents of e-cigarettes and analyze if there are any differences in the tweeting behavior of proponents as compared to regular tweeters	Feature Extraction - Feature groups explored from individual's bio and latest tweets: Unigrams, Bigrams, Part of speech tags, Positive and negative sentiment scores (SentiWordNet 3.0), features extracted from topic modeling (LDA), Presence of specific terms in username such as vape, vapor, etc.<br><br>Sentiment analysis of proponent tweets – SentiWordNet 3.0	ACC, PR, RC, F1 score	Using the best feature combination, the model was able to predict the proponents of e-cigarette with a mean F1 score of 0.92. The percentage of proponents were 7.5% from first dataset and 4.3% from second dataset. The mean positive and negative scores were 0.92 and 0.01 for the proponents. Proponents tweeted two to five times more than regular tweeters. The major themes identified from content analysis of proponent tweets were: highlighting the positive aspects of e-cigarettes like availability of different flavors, their smoke-free aspects, their claims about its use in smoking cessation efforts and their perceived

					Proponent Classification Model based on LR classifier (4-fold cross validation)		reduction in harm as compared to traditional cigarettes.
Kostygina, 2016 [60]	Nicotine Use	Twitter	Total – 4.5 million tweets (October 2014, March 2015, April 2015)<br><br>Manually annotated – 5124 for relevance, 2670 for co-use of tobacco and marijuana vs tobacco use only tweets, 3000 twitter accounts for organic vs commercial tweets	Thematic analysis of LLC-related Twitter posts – to identify product preferences, (brand and flavor), behaviors (purchase and use context) and social norms (subculture frames and peer group references)	Relevance Classification (LLC relevant tweets vs non-relevant tweets) – NB, LR, SVM (linear) with L1-norm regularization (10-fold cross validation)<br><br>Content Classification (organic vs commercial tweets AND co-use of tobacco and marijuana vs tobacco use only) – SVM (10-fold cross validation)<br><br>Account metadata was used to identify if the user can be categorized as influencer or not to	ACC, PR, RC, F1 score	Classifier achieved F1 score of 0.96 for identifying LLC relevant vs non-relevant tweets (n=4,372,293). Classifier achieved F1 score of 0.92 for identifying organic vs commercial tweets and 0.99 for identifying co-use of tobacco and marijuana vs tobacco use only tweets. The results showed that a majority of LLA-relevant tweets were organic (99%) and contained references to marijuana LLC co-use in form of blunts (83%). About 17% of account users posting the LCC content were influencers. Influencers were 30% more likely to mention specific LCC brands and 33% more likely to post promotional messages. The results of topic-modelling were

					identify the reach of LLC related tweets<br><br>Keyword algorithms were used to assess marketing strategies to attract youth and vulnerable populations<br><br>Topic modeling - LDA		grouped into 4 major categories: product-related messages, marijuana references, smoking behavior, and normative and cultural context references.
Lazard, 2016 [61]	Nicotine Use	Twitter	Total - 872,544 tweets and retweets, out of which 240,578 were included in the final topic groups (March 24, 2015 – July 3, 2015)	Analyze online interactions to determine the thematic interests and trending topics from commercial and consumer conversations regarding e-cigarettes	Text Mining / Topic Identification – SAS Text Miner 12.1<br><br>(Text Parsing, Text Filter, Text Topic using expectation maximization (EM) algorithm)		126,127 tweets were sorted into eight unique topics. Out of these, five topics included individual user conversations related to – e-cigarettes as cessation devices, pro-vaping reactions to e-cigarette policies, e-cigarette news and updates from proponents, vaping advocacy comments from proponents, and use of e-cigarettes by employees as a relief from smoking bans.<br><br>114,451 retweets were sorted into 5 unique topics out of which one topic consisted of proponent and individual user conversations related to discussion of policies banning e-cigarette use, e-

							cigarettes as cessation devices, and differentiation of e-cigarettes from traditional cigarettes.
Chen, 2015 [62]	Nicotine Use	Three online forums - Vapor Talk Hookah Forum and Reddit	Vapor Talk (13,814 posts), Hookah Forum (17,761 posts), and Reddit (134,712 posts) (April 2014 – June 2014)	Understand the experience of members using three tobacco products – combustible cigarettes, e-cigarettes, and hookah. Compare e-cigarette and hookah use behaviors and experiences across multiple forums to identify social and contextual factors influencing their usage such as settings, time, social relationships and sensory experiences	Lexicon development to identify contextual factors about e-cigarette and hookah use. It consisted to three categories – subject matter, health, and context.<br><br>Visualization – Heat maps to compare prevalence of contextual factors of e-cigarette and hookah use across different forums<br><br>Topic modeling - LDA<br><br>Topic modeling-based visualization - Topic Bars		The mentions about people, symptoms, time, quitting, and sensory experience were highest in density in the Vapor Talk Health & Safety forum and in the Stopsmoking subreddit. A total of 20 topics were generated from all subforums except for Hookah forum from which 40 topics were generated. In Vapor Talk Health, the two most prominent categories were Symptoms and Vaping versus Analogs. In the Stopsmoking subreddit, psychology and quitting methods were prominent topics. The prominent topics on the Hookah forum included experiential elements of use, and the buying and selling of equipment. The e-cigarette and hookah forums are similar as their members represent a “hobbyist

							culture" that actively engages in information exchange.
Cole-Lewis, 2015 [63]	Nicotine Use	Twitter	Total – 3.7 million tweets<br><br>Manually annotated – 17,098	Automate the categorization of e-cigarette-related content generated on Twitter into five categories: e-cigarette relevance, sentiment, member description, genre, and theme. The goal of the was study was to understand public's attitudes and beliefs regarding e-cigarettes to be able to inform public health interventions	Feature extraction- ranged from unigrams to 5-grams (n-grams), TF-IDF<br><br>Classifiers - NB, KNN, and SVM (linear)<br><br>(10-fold cross validation)	ACC	All manually coded tweets were used to build classification model to distinguish relevant vs non-relevant tweets. A total of 10,128 tweets were manually labeled as relevant and were used to build classification models for sentiment, user description, genre and theme. All classifiers performed best using linear SVM algorithm except for one category (Other substances) where KNN performed better. The most appropriate word-grouping unit (n-gram) was unigram for most of the classifiers. Models accuracy for predicting the various categories varied from 68.40% and 99.34%. The highest accuracy score along with highest percentage of possible improvement over random baseline was achieved for the following categories: Policy/Government (0.94, 80.64%), Relevance (0.94,

							75.26%), Ad or Promotion (0.89, 72.69%) and Marketing (0.91, 72.56%).
Myneni, 2015 [64]	Nicotine Use	QuitNet	Total – 16,492 messages (March 2007 – April 2007)<br><br>Manually annotated - 795	Identify, visualize, and analyze content-specific communication patterns pertinent to human behavior change in online settings. This will have implications for the design of socio-behavioral interventions based on social influence	Qualitative Analysis – Derived conversation themes and concepts through grounded theory techniques (open coding, axial coding, constant comparison)<br><br>Automated Analysis - LSA (using Semantic Vectors package)<br><br>SNA – Affiliation Network Analysis using 2-mode network graphs (UCINET)<br><br>Network autocorrelation model was generated using sna package in R to measure content specific communication patterns	Automated analysis – inter-rater reliability<br><br>2-mode network graphs – Degree centrality<br><br>Statistical analysis - p value <0.05 was considered statistically significant	A total of 12 communication themes were identified from qualitative analysis such as social support, QuitNet specific traditions, progress, cravings, etc. The average agreement of the automated system with human raters was 0.71. According to degree centrality metric, social support and cravings were the most prevalent themes embedded in the communication among QuitNet members. The members who were exposed to others who reported abstinence through group-centric interpersonal themes were more likely to stay abstinent themselves (b = 0.038; P < .01). The members who were exposed to others who reported abstinence through individual-centric interpersonal themes were more likely to be abstinent themselves (b = 0.078; P < .05).

Myneni, 2013 [65]	Nicotine Use	QuitNet	Total – 16,492 messages (March 2007 – April 2007)<br><br>Manually annotated - 100	Content Analysis of messages exchanged between the members of an online smoking cessation community	Qualitative Analysis – Derived conversation themes through grounded theory techniques (open coding, axial coding, constant comparison)<br><br>Automated Analysis - Word vector representations through utilization of TASA corpus to generate QuitNet message vector representations, LSA was used to recognize meaningful relationships between terms (Semantic Vectors package)<br><br>SNA – 2-mode them-based social networks created using Gephi	Automated Analysis – PR, RC<br><br>SNA – degree, modularity	A total of 9 themes were generated from the qualitative analysis like personal experience, social togetherness, advice, reinforcement, support, conflict, adherence, self-efficacy, and returns. These themes were consistent with various behavior change theories - Social Cognitive Theory, the Transtheoretical Model of Change, and the Health Belief Model. For automated analysis, the recall of the system was 0.75 and the precision was 0.81. Social network analysis revealed that high-degree nodes represent the opinion leaders of the network for that specific content.
Myslín, 2013 [66]	Nicotine Use	Twitter	Total – 7362 tweets<br><br>(December 2011 – July 2012)	Perform content analysis of tobacco-related tweets to capture their genre, theme, and sentiment. To automatically filter out irrelevant content from Twitter data in	Manual Content Analysis – All tweets were categorized into three categories: genre, theme and sentiment	ACC, PR, RC, specificity, F1 score<br><br>Statistical analysis - p value <0.05	As per manual content analysis only 4215 tweets were relevant and out of these most prevalent genres were first- (40%) and second-hand experience (14%) and opinion (9%). The most prevalent themes were

			Manually annotated - 7362	order to improve the signal-to-noise ratio. To Demonstrate the utility of Twitter in addressing public health challenges related to tobacco usage.	Inter-category Correlations – chi-square statistics<br><br>For Automated classification:<br><br>Feature Extraction – unigrams, bigrams and trigrams<br><br>Feature selection based on information gain<br><br>Classifiers - NB, KNN, and SVM (10-fold cross validation)	was considered statistically significant	hookah (20%), cessation (14%), and pleasure (11%). Overall, the tweets expressed positive sentiment (46%) towards tobacco smoking. Social relationships emerge as a key component of positive sentiment toward tobacco on Twitter. The highest inter-category correlations were observed between (1) underage usage and social image (0.6) and (2) e-cigarettes and marketing (0.54).<br><br>SVM with unigram features achieved highest F1 score (0.85) for discriminating tobacco-related tweets from unrelated tweets.
Sofean, 2013 [67]	Nicotine Use	Twitter	Manually Annotated - 500	Analyze opinions of individuals towards smoking, perform sentiment analysis of smoking-related tweets.	Feature Extraction – baseline (without preprocessing the tweets), unigrams, bigrams, unigrams + bigrams<br><br>Classifier - SVM using different feature sets (Weka)	ACC, RC, PR, F1 score	The highest F1 score of 85.5% was achieved with the baseline feature set and combination of both unigrams and bigrams.

					(5-fold cross validation)		
Kim, 2020 [68]	Drug / Substance Abuse	Twitter	Total – 34,293 tweets (August 2018 – July 2019)<br><br>Manually annotated - 6860	Analyze tweets about nonmedical use and side effects of methylphenidate (a prescription drug commonly used for treating attention deficit hyperactivity disorder)	Tweets were annotated for first-experience use and then those tweets were further classified into non-medical use, side effects and others. Finally, tweets were labeled as either positive for non-medical use or side effects and negative<br><br>Feature extraction – personal noun, non-medical use terms, medical use terms, side effect terms, sentiment scores, and presence of URL<br>(using tm package in R, Liu and Hu opinion lexicon dictionary)<br><br>Classifier – SVM (radial basis function kernel) using e1071 package from R and RStudio<br>(10-fold cross validation)	PR, RC, F1 score, ACC	The F1 score of SVM classifier for non-medical use was 0.547 and 0.733 for side effects. The low recall (0.388) of the classifier for nonmedical use was responsible for low F1 score even though it had high precision (0.926). The low F1 scores may have induced underestimation of nonmedical use and side effects in the test dataset (1.3% and 1.9%, respectively).

Nasralah, 2020 [69]	Drug / Substance Abuse	Twitter	Total - 502,830 tweets (June 2018 – April 2019)<br><br>Manually Annotated - 1000	Develop a text mining framework to evaluate data quality and analyze the user-generated social media content in order to identify themes and topics related to drug abuse	Creation of drug-abuse ontology to retrieve relevant tweets<br><br>Evaluation of data quality – Search query-based classifier (Classifier 1) and Evaluation Matrix based classifier (using NLTK package) (Classifier 2)<br><br>Topic Modeling – LDA	PR, RC, F1 score, ACC	According to the evaluation matrix, 366,736 tweets out of the total collected tweets were deemed relevant with a F1 score of 0.95. 18 topics in total were identified related to opioid use such as opioid crisis, chronic pain medications, how U.S. government deals with it, opioids drugs coming across the U.S. border, deaths because of overdose due to opioid fentanyl and heroin, opioid treatments, opioid crisis as a real problem, taking opioid medications for health problems such cough, opioid overdose deaths, opioid crisis impact on American communities, and patients suffering from opioid addiction.
O' Connor, 2020 [70]	Drug / Substance Abuse	Twitter	Total – 16,443 tweets<br><br>Manual annotation was done in sets of 4 (15,405, 8016, 6096,	Preparation of annotated dataset to detect prescription medication abuse detection and illustrate the utility of the dataset by comparing the performances of various supervised ML classifiers	Annotated Corpus Generation – creating a list of medications for study, keyword search for retrieval of tweets, labeling the tweets into 4 categories: potential abuse or misuse, non-abuse consumption, drug	F1 score, ACC, 95% CI for accuracy	The prescription medication abuse is discussed on Twitter in various ways such as including expressions indicating co-ingestion, nonmedical use, nonstandard route of intake, and consumption above the prescribed doses. The SVM classifier achieved highest F1

			6904 tweets respectively)		mention only, and unrelated using annotation guidelines following iterative annotation process<br><br>Feature extraction – n-grams (1-3) for regular classifiers word embeddings for d-CNN (word2vec)<br><br>Classifiers – NB, RF, SVM, d-CNN		score for abuse (0.53), consumption (0.67) and mention categories (0.82). For unrelated category, NB classifier achieved highest F1 score (0.81). SVM gave an overall accuracy of and 73% (95% CI 71.4-74.5) over the test set.
Desrosiers, 2019 [71]	Drug / Substance Abuse	Facebook	Text messages from phone were collected through Mobilespy software<br><br>Facebook posts were extracted using NVivo	Understand how affectivity (positive and negative) and stress contribute to substance use severity amongst ethnic minority adult males. Explore correlations between positive and negative affect indices, stress and substance use severity and understand if those affects relate to substance use severity	Analyze textual posts to categorize into thematic and linguistic categories – LIWC posemo affective category (captures positively valenced words such as love, nice, sweet) negemo affective category (captures negatively valenced words such as ugly, hate, kill)	Statistical analysis - p value <0.05 was considered statistically significant	The participants post provided a high total word count for linguistic analysis for each individual (M = 2,177.57; SD = 3,749.12). The most frequently used substances were marijuana, alcohol, ecstasy, prescription drugs, cocaine, mushrooms and LSD. The higher negative affect in posts was associated with greater substance use at 6-month follow up (r = .433, p < .05). Stress was associated with greater substance use at 6-month follow up (r = .417, p < .05).

					Correlation Analysis – GEE (to explore whether positive and negative affect predicted substance use severity and if stress moderated these associations)		
Hassanpour, 2019 [72]	Drug / Substance Abuse	Instagram	Images – 466,227 Captions – 369,000 Comments – 475,000	Identification of substance use risk based on social media posts on Instagram using deep learning technology using image and text classification techniques	Crowdsourcing platform used – Clickworker<br><br>Classifier<br>Image data: CNN ResNet18 model pre-trained on ImageNet data repository<br>Textual data – word2vec (pre-trained on Wikipedia corpus) & LSTM<br><br>Average feature vector was generated by combining Feature Vectors in joint image-text embedding space<br><br>Risk estimation model-fully connected neural network layer with softmax normalization	RC, PR, F1 score, AUROC	The model was able to estimate the risk of alcohol abuse with statistical significance (p-value = 0.00008). The deep-learning model for alcohol risk estimation achieved AUROC of 0.65 for images, captions and comments combined compared to baseline model (LR) which achieved AUROC of 0.55 for all three features combined.

					and a cross-entropy loss function<br><br>Optimization of neural network weights on training set (80%), hyperparameter tuning on validation set (10%) and evaluation on test set (10%)<br><br>Deep learning model optimized through SGD (Stochastic Gradient Descent)<br><br>Baseline Model - LR		
Hu, 2019 [73]	Drug / Substance Abuse	Twitter	Total – 3,265,153 tweets (January 2017 – February 2017)<br><br>Manually annotated – 1794 (seed dataset) and	Analyze tweets to identify drug abuse risk behaviors (positive) tweets as well as negative tweets from a highly imbalanced drug abuse dataset	Data annotation - used seed tweets (n=1794) to train SVM classifier, ran the trained classifiers on unlabeled dataset, posted 5000 of those to AMT crowdsourcing platform<br><br>Pre-training – word-level CNN was pre-trained on Drug Chatter word	ACC, RC, PR, F1 score	Deep learning ensemble model had better performance compared to traditional ML models. Within the deep learning models - C-CNN (character-level) model performed better than the W-CNN model (word-level). The F1 score for 50:50 class split for ensemble CNN model and ensemble traditional ML model was 0.85 and 0.86 respectively. The F1 score for

			1000 (AMT dataset)		embeddings (400 dimensions)<br><br>Features extracted - abuse-indicating term features, drug-slang lexicon features, synonym expansion features using WordNet, word cluster features<br><br>Predictive Model – ensemble deep learning model consisting of word level<br>CNN (W-CNN) with auxiliary features and char-level CNN (C-CNN) with and without auxiliary features<br>(6-fold cross validation)<br><br>Baseline ensemble model consisting of traditional ML classifiers - SVM, RF, NB		10:90 class split for ensemble CNN model and ensemble traditional ML model was 0.54 and 0.50 respectively.
Sarker, 2019 [74]	Drug / Substance Abuse	Twitter	Total – 131,000 tweets	Develop and evaluate text processing pipeline for	Feature Extraction – n-grams, word clusters and presence or	PR, RC, F1 score	The proportion of tweets belonging to different categories based on manual

			(January 2012 – October 2015)<br><br>Manually annotated – total of 9006 out of which 550 tweets were done by two raters	automatically characterizing opioid-related chatter into 4 categories - self-reported abuse, information sharing, non-English, and unrelated and combine the outputs with geospatial and temporal analysis	absence of abuse-indicating terms<br><br>Word embeddings (word2vec) used for deep learning classifier (domain-specific)<br><br>Under sampling of majority class and oversampling of minority class was done (SMOTE)<br><br>Classifiers - NB, DT, KNN, RF, SVM, deep CNN<br>(10-fold cross validation)<br><br>Geospatial analyses – social media post rates were compared to two reference datasets - WONDER database and NSDUH (National Survey on Drug Use and Health)<br><br>Correlation metrics used - Pearson and Spearman	For statistical analysis two-tailed $P < .05$ was interpreted as statistically significant	annotation was – 19.4% were abuse-related, 22.2% were informational posts, 4.7% were not English language, and 53.6% were unrelated to opioids. In terms of classifiers, SVM and RF achieved similar F1 scores – 0.700 and 0.701 respectively. D-CNN achieved F1 score of 0.72. Statistically significant correlation (Pearson r = 0.451, P < .001; Spearman r = 0.331, P = .004) was found between the county-level overdose death rates and the abuse-indicating social media posts over 3 years.

Sarker, 2019 [75]	Drug / Substance Abuse	Twitter	Total - 9006 tweets were manually annotated, out of which 550 were done by 4 annotators	Real time surveillance of opioid abuse/misuse by performing content analysis of tweets mentioning prescription and illicit opioids. To compare the distribution of prescription and illicit opioids tweets using 4 broad categories - self-reported abuse, information sharing, non-English, and unrelated	Feature Extraction – n-grams (1-3), word clusters and presence or absence of abuse-indicating terms<br><br>Word embeddings (word2vec) used for deep learning classifier (domain-specific)<br><br>Classifiers – SVM, RF, deep CNN, NB (baseline)<br>(10-fold cross validation)	PR, RC, ACC	The annotated data set consisted of 1748 abuse tweets, 2001 information tweets, 4830 unrelated tweets, and 427 non-English tweets. The dataset consisted of about 75% tweets mentioning illicit opioids. And 25% mentioning prescription opioids. For both prescription and illicit opioids, a significant proportion of tweets were unrelated to opioids which suggested higher amounts of noise associated with such tweets. Prescription opioid mentioning tweets had higher proportions of misuse/abuse and information-oriented tweets. In terms of classification, deep CNN gave the best performance with an overall accuracy of 70.4% which was very close to other classifiers (RF -70.1%, SVM – 69.9%).
Glowacki, 2018 [76]	Drug / Substance Abuse	Twitter	Total – 73,235 tweets (August 2016 –	Identify topics and extract meanings contained in unstructured textual data in order to understand public's	Text Mining / Topic Identification – SAS Text Miner 12.1<br><br>(Text Parsing, Text Filter, Text Topic using		Seven mutually exclusive topics emerged as the most popular from the set of original tweets, and 13 mutually exclusive topics emerged as the most popular

			October 2016)	reaction to opioid abuse	expectation maximization (EM) algorithm)<br><br>NUVI software was used to aggregate information such as trending hashtags, top influencers, locations of tweet, etc.		from the set of retweets. Example topics: calls for treatment options for teen addicts, the promotion of marijuana as an effective alternative for managing pain, concerns about the double standards in care when treating black and white opioid users, the positive effects of using opioids, etc. The Associated Press (AP) emerged as the biggest influencer on Twitter. California, Kansas and New York had highest number of overall opioid mentions. Washington DC, Kansas, and New York had the largest concentration of people tweeting about opioids per capita.
Graves, 2018 [77]	Drug / Substance Abuse	Twitter	Total - 84,023 tweets (January 2012 – April 2012, April 2013 – October 2015, July 2009 – February	Identify topics related to opioid discussion on Twitter and correlated those topics various geographical regions as well as opioid overdose death rate. Understand the misuse patters in different geographical areas.	Topic Modeling – LDA<br><br>Correlation between topics and opioid death rate – differential language analysis, open-vocabulary approach, and Pearson Correlation Coefficient		Drug-related crime theme was more common in the northeastern divisions, language of use theme was more common in the southeastern divisions. Some other themes identified were addiction, addiction treatment, opioid-related news, overdoses, pop culture etc. The topic most

			2011, April 2012 – April 2013)				correlated with opioid overdose death rate at the county level (r = 0.331) contained the words “police,” “arrested,” “trafficking,” “bust,” and “dealer”, belonging to the theme of “drug-related crime.” Other topics that significantly correlated with opioid overdose death rate at county level were opioid-related news (r=0.278) and pop culture (r=0.282). The topic correlated with opioid overdose death rates at the state level (r = 0.449) included the words “prescription,” “online,” “delivery,” “cheap,” and “buy”, belonging to the theme “online drug purchasing.”
Kalyanam, 2017 [78]	Drug / Substance Abuse	Twitter	Total – 11 million tweets (June 2015 – November 2015)	Identify tweets relevant to NMUPD by increasing the signal to noise ratio in the dataset of all tweets. Identify themes and patterns in order to gain a broader understanding of NMUPD behaviors for	Pattern / Theme Recognition - BTM (unsupervised ML approach)<br><br>The themes were discovered for three prescription opioid analgesic	Evaluated the quality of themes using two approaches<br><br>1) Supervised evaluation by	The percentage of tweets retained after the second and the third iteration rounds was 72%–84% which led to approximately 2.3M identified tweets that were relevant to NMUPD. The results showed that polydrug abuse is predominantly associated with Twitter

				a larger Twitter member population.	drugs – Oxycontin, Oxycodone, Percocet	manually annotating tweets for each theme and calculating average false positive rate<br>2) Unsupervised evaluation by calculating cluster purity that quantifies how coherent the theme is.	prescription drug abuse discussions. This could be indicative of larger behavioral trends of members abusing multiple prescription drugs. Another NMUPD behavior detected was use of street or slang terms associated with polydrug abuse combinations or drug abuse related behaviors. The average false positive rate for Percocet, Oxycontin, and Oxycodone was 55%, 28%, and 14% respectively. The average cluster purity across themes for Percocet, Oxycontin, and Oxycodone was 0.43, 0.57, and 0.67 respectively.
Meng, 2017 [79]	Drug / Substance Abuse	Twitter	Total - 688,757 tweets	Sentiment analysis of substance use, identification of popular items associated with	Geocoding the tweets - GIS Python libraries (Shapely, Rtree, and Fiona)	Agreement between human and computer labeling	Alcohol (n=638,347) was the most commonly tweeted substance as indicated by most widely used terms such as “beer”, “drunk” and

			(April 2015 – March 2016)	substance use and most commonly tweeted substance terms among adolescent and young adults.	Tweet Processing – PTBTokenizer<br><br>Sentiment score for each tweet - Maximum Entropy Text Classifier (MALLET) to classify tweets into positive or negative sentiments<br><br>Extraction of Alcohol, smoking, and drug-related mentions - Maximum Entropy Text Classifier (MALLET)<br><br>Underage tweets - Maximum Entropy Text Classifier (MALLET)<br><br>Statistical Analysis - Linear regression models to examine the associations between Twitter-derived indicators for substance use and socioeconomic	was calculated<br><br>Statistical analysis - p value <0.05 was considered statistically significant	“cocktail”. The second most tweeted substance was marijuana. The majority of underage tweets were also about alcohol. In terms of sentiment analysis, 34.1% of substance use tweets were classified as happy. The percent of happy tweets ranged from 27% to 48% for alcohol, 9% to 54% for smoking, and 7% to 35% for drug use. The percent of happy tweets ranged from 27% to 48% for alcohol, 9% to 54% for smoking, and 7% to 35% for drug use.

					characteristics at the zip code level (STATA)		
Daniulaityte, 2016 [80]	Drug / Substance Abuse	Twitter	Total - 15,623,869 tweets<br><br>Manually annotated - 4000<br><br>(eDrugTrends platform)	Analyze drug abuse tweets related to synthetic cannabinoid use to automatically classify them by their source/type of communication (personal, retail, media) and sentiment (positive, negative, neutral)	Feature extraction – unigrams, bigrams, TF-IDF<br><br>Feature selection – chi-square test<br><br>Classifiers - LR, NB, and SVM<br><br>Source Classification Models:<br>Approach 1 (use all tweets) - Multiclass classification for all three source types followed by binary classification (using the best performing classifier from multiclass classification)<br>Approach 2 – use expanded URL's and followed same steps as approach 1<br><br>Sentiment Classification Models:	PR, RC, F1 score<br><br>Statistical analysis - p value <0.05 was considered statistically significant	The study results showed that supervised ML methods can be used with high accuracy to classify cannabis- and synthetic cannabinoid–related tweets. For classifying tweets by source or type, SVM that used expanded URLs produced best results (F1 score - 0.8102). For sentiment classification, the SVM algorithm that focused on personal communication tweets, performed better than other approaches (F1 score – 0.88). SVM performance was over 30% better compared with VADER (F=0.5116), and the difference was statistically significant at P<.001.

					Approach 1 (use all tweets) - Multiclass classification for all three sentiment types followed by binary classification (positive vs negative)<br>Approach 2 – use personal communication tweets only and followed same steps as approach 1<br><br>VADER (lexicon and rule-based method) was also used to classify manually annotated tweets<br><br>Statistical Analysis – one-tail t-test to determine if differences in F1 scores for different classifiers is statistically significant as compared to VADER (5-fold cross validation)		
Sarker, 2016 [81]	Drug / Substance Abuse	Twitter	Total – 129,448 tweets	Automated classification of member posts to	Feature extraction – n-grams (unigram, bigram, trigram),	PR, RC, F1 score, ACC	The percentage of tweets containing medication abuse signals were as follows:

			Manually annotated – 6400 tweets<br><br>(March 2014 – June 2015)	identify prescription medication abuse tweets for three abuse-prone medications – amphetamine mixed-salts (Adderall), oxycodone (OxyContin), and quetiapine (Seroquel).	abuse-indicating term features, drug-slang lexicon features, synonym expansion features using WordNet, word cluster features<br><br>Classifiers – NB, SVM, Maximum entropy, J48 DT, and stacking (combining all 4 classifiers) (LibSVM, Weka tools were used) (10-fold cross validation)		Adderall (23 %), quetiapine (5.0%), oxycodone (12%). For classification task, weighted SVM achieved highest F1 score of 0.45 for abuse-related tweets while stacking technique achieved F1 score of 0.46 for abuse-related tweets. The most useful feature set was n-grams for abuse-related tweets with an F1 score of 0.42. The proportions of abuse-indicating tweets for Adderall and Oxycodone increase over the analyzed period of time thus indicating the presence of abuse for these specific medications.
Crocamo, 2020 [82]	Alcohol Use	Twitter	Total – 1,044,400 tweets<br><br>Collected in 3 sets - D1, D2, D3 (December 2017 – March 2018, April 2018 – June 2018, July 2018 –	Explore the relationship between binge drinking (BD) and alcohol-related messages posted on social media by performing content analysis of the topics discussed.	Identification of genuine users based on meta-data and linguistic features (personal tweets) - number of tweets of a single user account, average number of hashtags per tweet, average number of mentions per tweet, n-grams (bigram, trigram) etc.	AUROC	For identification of genuine members (personal communication) SVM achieved AUROC values of 0.76 and RF achieved AUROC values of 0.73 on test set. The classification of potential binge drinkers achieved AUROC value of 0.67 with RF classifier on test set.

			September 2018)<br><br>Manually annotated - 500		Identification of potential binge drinkers based on linguistic features (alcohol-related behaviors) - TF-IDF score<br><br>Classifiers – SVM, RF (5-fold cross validation)		
Giorgi, 2020 [83]	Alcohol Use	Twitter	Total - 3.3 million tweets mapped to 3095 U.S. counties (June 23, 2009 – February 5, 2015)	Examine whether alcohol use related online interactions correlate with the county-level alcohol consumption rates, perform content analysis of binge drinking-related tweets, examine the regional and cultural differences in the language of those tweets, and characterize the content of such tweets using linguistic features	Topic Modeling – LDA (Gibbs sampling)<br><br>Differential Language Analysis – to characterize the language used across high and low drinking counties (linear regression) and to identify language associated with different cultural communities (logistic regression)<br><br>Sentiment analysis – To measure the relationship between sentiment and personal pronouns:	Statistical significance level of .05 was used for differential language analysis	Drunk topics most associated with high levels of drinking at the county levels were related to drinking and partying with friends (r=0.20), drinking with family (r=0.15), DUI (r=0.14), and falling both asleep and down the stairs.<br><br>In terms of cultural differences, religious communities had a high frequency of anti-drunk driving tweets, Hispanic centers discussed family members drinking, and college towns discussed sexual behavior. In terms of sentiment analysis, positive sentiment tweets tended to

					LIWC was used to measure personal pronoun use within each community, National Resource Council (NRC) Hashtag Sentiment Lexicon was used to measure the positive sentiment associated with a tweet		be more impersonal whereas tweets containing negative sentiment tended to be more personal in nature by carrying more personal pronouns.
Jelodar, 2020 [84]	Alcohol Use	patient.info/forums	32273 patient-questions from an alcohol forum of patient.info (June 2006 – January 2019)	Present a sematic framework for mining healthcare text documents for information retrieval and knowledge discovery to identify topics of discussion focusing on healthcare issues related to alcoholic beverages.	Topic Modeling for semantic mining – LDA (Gibbs sampling)<br><br>Semantic topic classification – RF, KNN, SMO (Sequential Minimal Optimization), MP (Multilayer Perceptron) (Weka)	ACC, MCC (Matthew's correlation coefficient) AUROC, F1 score	The authors were able to identify ten most important topics as well as ten most worthless topics of discussion among individuals of the forum. One such important topic refers to members explaining their experiences or questions regarding "Alcohol withdrawal and Detox" containing words such as alcohol, day, drinking, detox, job, etc. For sematic topic classification, LDA+RF model gave the highest performance (F1 score – 0.988).
Parackal, 2020 [85]	Alcohol Use	Facebook	819 comments	To identify plurality of topics and sentiment (polarity) associated with the comments of a	Text Mining - Text Miner add-on to SAS Enterprise Miner version 14.1		Nine different topics were identified related to alcohol consumption – baby's health, no safe amount, stop acts

			(June 2015 – September 2015)	health promotion campaign. This study analyzed the comments generated from 'Don't know? Don't drink' campaign conducted encourage women to stop drinking for a likely future pregnancy	Sentiment Analysis – SentiWords lexicon<br><br>Statistical Analysis - To investigate the association between topics and their sentiment linear regression analysis was done		deemed risky, safe sex, not worth the risk, good campaign, drinking nights, missed a pill, and girl in the ad. There were three topics that were associated with both positive and negative sentiment ($p<0.0001$) – baby's health, no safe amount, and safe sex which confirmed the polarity of sentiment even at the topic level.
Cohn, 2019 [86]	Alcohol Use	BecomeAnEX.org	Total – 814,258 posts<br><br>(January 2012 – May 2015)<br><br>Manually annotated – 1850 posts to determine if they contained reference to alcohol use	Understand the impact of alcohol use during an attempt to quit smoking by analyzing the discussions from an online smoking cessation community. To identify member characteristics associated with alcohol-related topics, examine distribution of negative sentiment towards alcohol use during quit attempt, understand the impact of member's social network centrality on their conversation topics	Identification of alcohol-related content – Using manually annotated posts four ML classifiers were trained (10-fold cross validation)<br><br>Topic Modeling – CTM (unsupervised technique) was performed on 6095 posts identified from previous step containing reference to alcohol use (Used "topicmodels" package in R)	Identification of alcohol-related content – ACC, F1 score, AUROC<br><br>SNA – in-degree and outdegree centralities	Out of the total 10 discussion topics that were discovered, three most salient themes were: 1) cravings and temptations around alcohol; 2) similarities of nicotine addiction to alcoholism; and 3) celebratory discussions of quit milestones including "virtual" alcohol use and toasts. From the posts containing the three most relevant topics, only 35% of the total posts expressed negative sentiment, but the distribution of sentiment differed by topic ($p<0.001$). Overall most of the topics did not have a negative sentiment about alcohol use.

					Feature extraction for sentiment analysis – meta-features, unigrams weighted with TF-IDF and their distances to alcohol keywords<br><br>Sentiment Analysis - J48 DT, AdaBoost with NB<br>weak learners, NB with under-sampling, RF with under-sampling<br><br>SNA – NetworkX package (Python)<br><br>Correlation Analysis – GEE using SAS software (to examine the association between social network centrality and topics)		The topics of discussion for an individual varied as per their social network connectivity (p<0.001 for topics - cravings and temptations around alcohol and similarities of nicotine addiction to alcoholism). The posts about cravings and temptations around alcohol were roughly 2.5 times more frequent among members with low-to-medium connectedness than members with high connectedness, posts about alcohol for celebrations) were most common among highly connected members.
ElTayeby, 2019<br>[87]	Alcohol Use	Facebook (Group – "I'm Shmacke d")	Total – 4266 posts<br><br>(November 2014)	To determine the feasibility of using social media data for detecting drinking-related posts. Perform automated classification of social media posts to identify	Manual content annotation performed to classify posts into three categories – yes, no, maybe<br><br>Automated Text Classification:	PR, RC, F1 score	It is feasible to automate the detection of drinking-related posts on social media using text and image/video classification techniques. The performance of SVM (linear) was highest on the text classification task (F1 score -

				drinking-related content amongst college students using heterogenous data types – using text, image and video content.	Feature Vectors created with TF-IDF<br><br>Classifier – SVM (with linear, polynomial, sigmoid, and radial basis function kernels), LLDA (supervised topic model using two algorithms – Gibbs sampling and collapsed variational Bayes with a zero-order Taylor expansion approximation (CVB0))<br><br>Automated Image/Video Classification: Neural Network (AlexNet with Nestrov's accelerated gradient solver)		0.72) as opposed to LLDA (F1 score - 0.69). The F1 score for AlexNet was 0.38 and 0.88 for image and video classification respectively. Combined SVM model (text, image, video) achieved the F1 score of 0.71.
Golbeck, 2018 [88]	Alcohol Use	Twitter	Total 225 users included in the study for whom recovery/relapse data at	Predict whether alcoholics will achieve and maintain sobriety before they begin treatment by analyzing their online social media interactions. It	Psycholinguistic text analysis – LIWC<br><br>Feature Extraction – psycholinguistic features, behavioral information features,	ACC, PR, RC, AUROC, F1 score	The linguistic features identified were affect, insecurity, and drives (including goal orientation). The classifier achieved an overall accuracy of 85% and an overall AUROC of 0.815.

			90 days was available (collected their most recent 3200 tweets from January 2013 – November, 2015)	is based on predicting people's future behavior based on their past actions by developing a feature set containing data about user's social connections, language, and psychological attributes.	age, social factors (such as the frequency with which they used alcohol-related words and the percentage of friends who tweeted about friends), coping styles (adaptive, maladaptive)<br><br>Classification Task – to determine whether users "recovered" or "not recovered" from alcoholism at 90-day mark<br><br>Classifier – REPTree (Weka)<br><br>(10-fold cross validation)		Thus, using the features used in this study, one can accurately estimate the predictors of alcohol recovery by automatically processing online social media interactions.
Kornfield, 2018 [89]	Alcohol Use	Peer-to-Peer Online Discussion Forum which is a part of smartphone application called -	Total -1625 messages	Predict the likelihood of relapse amongst individuals treated for alcohol use disorder (AUD) by analyzing various psychological traits and states characteristic of recovery manifested through language used by individuals in an	Language modeling – Extract linguistic features of AUD discussion boards through LIWC software. Linguistic correlates of relapse included affect, cognition, social integration, personal concerns.		The final model including linguistic cues was able to predict relapse with an accuracy rate of over 80%. The results of the study showed that the negative affect indicators in form negative emotions or swear words, inhibition words, and love words were significantly associated with increased

		Addiction – Comprehensive Health Enhancement Support System (A-CHESS)		online discussion forum	System log measures were also recorded as additional covariates such as number of messages posted, percent of days members logged into the forum, etc.<br><br>Assess the extent to which language features might predict likelihood of relapse (Predictive Modeling) - LR model		relapse risk. Cognitive mechanism words, death words, and increases in achievement were associated with decreased relapse.
Carah, 2017 [90]	Alcohol Use	Hello Sunday Morning blog		Analyze the blog posts of "Hello Sunday Morning" to understand how participant's expression changes over time regarding alcohol use.	Visual Text Analysis - Leximancer		During the first month, participants set their goals to quit alcohol consumption, discuss their anxiety towards the quit process. After the first month, the participants discussed their achievements, the challenges they faced during the quit attempt.
Cohn, 2017 [91]	Alcohol Use	BecomeAnEX.org (EX)	Total – 814,258 posts<br><br>(January 2012 – May 2015)	Analyze the content and sentiment associated with alcohol use in a smoking cessation community. The four content domains were -	Feature extraction – meta features, unigrams, bigrams, TF-IDF score<br><br>Classifiers – used different classifier for	Automated classification – ACC, F1 score, AUROC	The F1 scores of 0.86, 0.81, 0.95, and 0.81, were achieved for the 4 content domains using J48 DT, AdaBoost with NB weak learners, NB with under-sampling, and RF with under-sampling respectively.

			Manually annotated – 1850 posts to determine if the posts belonged to specific content domain	identify the presence of alcohol mentions in the post (CD1), identify whether alcohol-related posts are associated with actual personal use (CD2), identify whether the individual is referring to himself in problem drinking post (CD3), identify whether the sentiment about alcohol is negative as related to quit attempt (CD4). Also, to identify if the sentiment about drinking during quit attempt associated with social network position.	each of the four content domains (J48 DT, AdaBoost with NB weak learners, NB with under-sampling, RF with under-sampling) (10-fold cross validation)<br><br>SNA – constructed a directed network to visualize user's reading and posting behaviors<br><br>Statistical analysis - Univariate analysis of variance tests to examine whether expressing negative sentiment about drinking was associated with higher or lower levels of network connectivity	SNA - in-degree and out-degree centralities	The results of the classification showed that alcohol-related posts represented just under 1% of all posts made during the study period from which 33.02% described personal experience, and only 3.65% were related to problem drinking. About 33.07% of alcohol related posts contained negative sentiment. Social network analysis results showed that individuals who expressed negative sentiment towards alcohol had greater centrality both in-degree and out-degree.
Liu, 2017 [92]	Alcohol Use	Twitter	2 datasets<br><br>1) 804,000 tweets (June 2015)	Detect relevant alcohol-use behavior at high temporal resolution utilizing social media posts. Assess population-level temporal trends	Hierarchical Classification – First identify alcohol-related tweets, from these identify first-person use tweets, and these were classified	F1 score, AUROC	"Looking to drink" stage of alcohol behavior had the highest probability from Tuesday through Friday and was identified with highest F1 score of 0.64 through LR. "Currently drinking"

			2) 4,839,870 tweets (December 2015 – January 2016)	of high-resolution behavior stages of drinking alcohol. Examine behavior stage trends stratified by groups and at high-risk time periods.	into three behavioral stages – looking to drink, currently drinking, and reflecting on alcohol consumption.<br><br>Labeling – AMT and Active Learning<br><br>Feature extraction – n-grams, user features, TF-IDF features<br><br>Classifiers – LR, SVM, and RF<br>(5-fold cross validation)		behavior stage had the highest probability over the weekend and was identified with highest F1 score of 0.72 through LR. "Reflecting about drinking" had the highest probability from Monday through Wednesday and was identified with highest F1 score of 0.53 through LR. There were differences in alcohol use patterns among the different genders.
Parackal, 2017 [93]	Alcohol Use	Facebook	819 comments (June 2015 – September 2015)	Examine user engagement with 'Don't know? Don't drink' campaign conducted to encourage women to stop drinking for a likely future pregnancy. Analyze the comments generated by users to identify various themes and sentiments.	Text Mining - Text Miner add-on to SAS Enterprise Miner version 14.1<br><br>Statistical Analysis - To investigate whether the contents of the message were related to or predicted by the comments using Logistic Regression	Statistical significance level of .05 was used for logistic regression	Four themes were identified during text mining phase – risk of pregnancy, alcohol and culture, credibility of the campaign, and contraception failure. The results of logistic regression showed that the base level likelihood of the message being featured in the comments was comparatively small and the overall probability of a message receiving any reaction was about 70%. The

					Sentiment Analysis – Mathematica 10.3 was used to categorize messages into positive, negative and neutral		results of sentiment analysis showed that the campaign generated all three sentiments but the proportions of negative comments were higher than positive and neutral comments.
Cesare, 2019 [94]	Physical Activity	Twitter	Total - 1,382,284 geo-tagged tweets (April 2015 – March 2016)	Identify types of physical activities in which members engage and the intensity of those activities. Also, identify if there are any association between physical inactivity tweet patterns and physical activity prevalence varied by different geographical regions and by sex.	Classifiers for identifying physical activity tweets - feed-forward neural network, SVM, gradient boosting and fastText, keyword matching algorithm<br><br>Sentiment Analysis – Maximum entropy text classifier, training sets obtained from Kaggle, Sentiment140 and Sanders Analytics<br><br>Sex classification – ensemble classifier based on weighted stacked LR framework using multiple features extracted from user's names	RC, ACC, F1 score<br><br>Statistical significance level of .05 was used	For identification of physical activity related tweets, the keyword matching algorithm achieved performed the best with F1 score of 0.90. For sex classification, the ensemble classifier achieved F1 score of 0.84. Overall men and women shared similar sentiment towards physical activity (score-0.66 and 0.657 respectively). The results of the study showed that men engaged more in higher intensity physical activities than women. Walking was the most popular physical activity for men and women across all regions (15.94% and 18.74% of tweets, respectively). Men and women mentioned performing gym-based

					Statistical Analysis – linear mixed-effect regression models to examine the association between indicators of physical activity from Twitter data and county-level estimates of physical inactivity		activities at approximately the same rates (4.68% and 4.13% of tweets, respectively). Men mentioned engaging in higher intensity activities than women. Also, counties that reported higher levels of physical activity tended to have lower physical inactivity prevalence as per the CDC survey estimates.
Nguyen, 2016 [95]	Physical Activity	Twitter	Total - 80 million geotagged tweets<br><br>(February 2015 – March 2016)<br><br>Manually annotated – 5000	Build a national neighborhood database from data generated via Twitter in order to characterize well-being and various health behaviors. To explore the associations between Twitter-derived neighborhood variables and neighborhood demographic and economic characteristics.	Geocoding the tweets - GIS Python libraries (Shapely, Rtree, and Fiona)<br><br>Tweets were characterized by three variables – happiness, food, physical activity<br><br>Sentiment Analysis to identify happy and not happy tweets – Maximum Entropy Text Classifier (MALLET), training sets obtained from Kaggle, Sentiment140 and Sanders Analytics	ACC, FI-score	The performance of the ML algorithm was as follows: 78% for happiness, 83% for food, and 85% for physical activity with the F1 scores 0.54, 0.86, and 0.90, respectively. Overall, 20% of tweets were classified as happy, 5.1% of tweets were about food and 1.8% were about physical activity. Census tract characteristics such as percent African American (beta coefficient, B=−.11), greater household size (B=−.18), and economic disadvantage (B=−.19) were related to lower tract happiness. Happy tweets were more prevalent in zip codes with higher numbers

					Food analysis – Text-matching algorithm to identify food mentions in tweets, sentiment analysis to identify health foods and fast foods, identify caloric density in food mentions<br><br>Physical Activity analysis - algorithm created for identifying following variables from tweets – physical activity mention, exercise intensity, and sentiment towards physical activity<br><br>Evaluated sentiment analysis results through AMT, bag-of-words algorithm and Sentiment140<br><br>Statistical Analysis - Adjusted linear regression models were applied to examine associations between area-level Twitter		of businesses (B=.11) and full-service restaurants (B=.16). A large number of fitness and recreational sports centers were related to higher exercise intensity (B=.05) and happier tweets (B=.07) at the zip code level.

					characteristics and other characteristics		
Yoon, 2013 [96]	Physical activity	Twitter	Total - 174,394 tweets (March 2010 – May 2010)<br><br>Tweets imported via NodeXL	Analyze topics, sentiment and ecological context of tweets related to physical activity. To understand the content of tweets that mention specific physical activities, identify if that content varies by specific physical activity and the sentiment associated with those tweets.	Feature Extraction – n-grams (unigram, bigram, trigram)<br><br>Topic detection – calculated frequency vectors to create term-Tweet frequency table, performed chi-square test to compare terms across corpus<br><br>Visualized content change over time through 2D and 3D motion charts<br><br>Sentiment analysis – used a tool called twittersentiment.appspot.com (Sentiment140) to identify topics associated with positive and negative sentiment<br><br>Categorization of ecologic momentary context – Qualitatively analyzed for aspects		The computed Tweet term-frequency dictionary contained a total of 31,489 terms from the corpus of 174,394 Tweets. In terms of distinct content across physical activities, the term “good” appeared across all activities, whereas “obesity” occurred only for a few activities. Tweet categories associated with more than 40% negative sentiment were – walking fast, jogging, running, etc. Tweet categories consistently associated with positive sentiment were – hiking, golf, dancing, etc. Tweets also contained information about physical, emotional and social context.

					such as time, purpose, environment, social context and feeling		
Liu, 2020 [97]	Obesity-related behaviors	Reddit (r/loseit subreddit)	477,904 posts (16,332 submissions and 461,572 comments published before January 13, 2018)	Understand the extent to which content posted by users of r/loseit subreddit and their online interactions were associated with weight loss in terms of number of replies and votes that the individuals received.	Topic Modeling – LDA<br><br>Word Semantic Clustering – word2vec model<br><br>Regression Analysis – Linear regression model to understand the association between weight loss and online discussions using predictors such as – active days, number of posts, topic distribution, word semantic clusters, votes received by individuals for each post and median number of comments for each post published by an individual. The outcome variable for regression model was weight loss (start weight – current weight)	Statistical significance level of .05 was used for regression analysis	25 topics were discovered that were summarized into 11 categories - food and drinks,<br>exercise, calorie, clothes, time, health issues, weight change,<br>feelings, plans, and communication.<br><br>Start weight (β=.823; $P<.001$), active days (β=.017; $P=.009$), and median number of votes (β=.263; $P=.02$), mentions of exercises (β=.145; $P<.001$), and nutrition (β=.120; $P<.001$) were associated with higher weight loss. Individuals with higher weight mentioned experiencing negative emotions before they tried to lose weight. Some factors that were associated with lower weight loss included mentions of vacations and clubs, supermarkets, refund to exercise programs, health issues, and family members.

Liang, 2019 [98]	Obesity-related behaviors	Twitter	4010 tweets from Houston, 3281 tweets from San Diego (Aug 2015 – Aug 2016)	Examine Twitter content from two major U.S. cities to understand the differences in social environment of individuals regarding weight loss.	Text mining – tm package in R<br><br>Topic Modeling – LDA to identify topic related to three themes – weight loss, diet, and gym/fitness<br><br>z-test statistics was performed for comparing relative word frequencies between the two cities	Statistical significance level of .05 was used for z-test	San Diego users had higher proportion of gym/fitness related tweets and low proportion of diet-related tweets. The most popular topic related to gym/fitness theme for San Diego users was related to their capability of managing time. There was an association between mentioning of weight loss, dieting, and exercising in San Diego suggesting that such a "fitness" theme might encourage individuals to supplement exercise with dieting for weight control. While this association was not found for Houston users, indicating the people of Houston tend to link weight-loss behaviors with burning calories and dieting and viewed exercise separate from dieting behaviors.
Ghosh, 2013 [99]	Obesity-related behaviors	Twitter	Total - 455,981 geotagged tweets in total (October 2011 –	Identify topics and themes related to obesity on Twitter. Understand the spatial distribution of obesity related tweets.	Geocoding tweets using functions in ESRI's (Environmental Sciences Research Institute) ArcGIS 10.0		Three major themes were identified - childhood obesity and schools (4.79% of all tweets), obesity prevention (6.96% of all tweets), and obesity and food habits (9.68% of all tweets). The

			March 2012)		Text mining – tm package in R<br><br>Feature extraction – bigrams<br><br>Topic Modeling – LDA		spatial analysis of the extracted themes showed distinct pattern between rural and urban areas, northern and southern states, and coastal and inland states.
Shah, 2020 [100]	Physical Activity & Obesity-related behaviors	Twitter	Total – 99,999,986 tweets (2018 - 2019)	Analyze social media data in order to identify nutrition and physical activity related tweets in order to get health information about the health of Canadians	Binary classification to identify food vs non-food related tweets.<br><br>Feature extraction – TF-IDF with phrases (for traditional classifiers), word embedding with bigrams (with deep learning classifiers)<br><br>Classifiers – NB, LR, RF, SVM SNN, CNN, Reinforcement neural network (RNN-GRU)<br><br>Information about calories and user's activity was obtained from various algorithms	ACC	RF model gave the highest accuracy for binary classification of tweets in food and non-food categories (93.4%) as compared to other classifiers. Among the top foods, junk food and hot drinks were most common. Watching something was the most common physical activity followed by reading, walking and running which indicates the rise of physical inactivity throughout Canada. The results showed that watching (TV) is the most common activity in dense populated provinces of Canada. The results of the study also showed rapid growth of obesity in Ontario and Quebec regions.
Park, 2018 [101]	Nicotine &	Reddit	Total - 114,320,798 posts, and	Understand whether Reddit is an effective source for identifying	Lexicon-based approach to extract relevant posts using		The results of the study showed that e-cigarettes and marijuana specific topics are

	Substance Abuse		1,659,361,605 associated comments<br><br>(October 2007 – May 2015)	controversial health related issues like usage of e-cigarettes and marijuana, and what are the kinds of topics are discussed amongst members of the community regarding these health issues	specific key terms, calculated normalized frequencies over time counts<br><br>Topic Modeling – LDA<br><br>Word Clouds for visualizing the main topics and their associated words		being discussed in many different subreddits. the normalized count on marijuana almost doubled in February 2009 and the discussions on e-cigarettes remained steady from October 2007 to May 2015. Members of the community created 244 new subreddits to discuss about marijuana and 3 new subreddits to discuss about e-cigarettes. Topics such as ‘legalization’, ‘prohibition’, ‘economy’, and ‘state’ appeared in discussions regarding marijuana. Topics such as ‘quitting smoking’, ‘fun’, ‘experience’, and ‘health information’. appeared in discussions regarding e-cigarettes. One subreddit called ‘Ecigclassifieds’ consisted mainly of commercial content. The e-cigarette subreddits also contained commercially related terms such as ‘quality’, ‘prices’, ‘shop’, and ‘store’.

Huang, 2017 [102]	Nicotine & Alcohol Use	Twitter	50 million tweets per month<br><br>(June 2015, September 2015)	Understanding tobacco- and alcohol-related behavioral patterns amongst twitter members. Explore the kinds of high frequency representations resolved from Twitter data that can be used to evaluate meaningful differences in such temporal patterns of communication.	Three kinds of classification were done – behavior vs not behavior, first person vs not first person, and past, present and future behavioral stages.<br><br>Feature extraction – unigrams, bigrams, trigrams<br><br>Labeling - AMT and Active Learning<br><br>For classification of alcohol and tobacco behaviors - LR, SVM and RF<br>(10-fold cross validation)<br><br>Age Classification for alcohol-related dataset - unigram features from the text with SVM (linear) classifier<br><br>Tobacco product identification – Keyword filtering	ACC, F1 score, AUROC	F1 scores for the behavior, first-person and present classifiers were: 0.87, 0.75 and 0.81 (alcohol) and 0.86, 0.98, 0.75 (tobacco). The F1 score of age classifier was 0.82. The results of the study showed that overall alcohol consumption differs by day of the week, as well as these trends vary on weekends versus weekdays. These trends are similar by week at different times of the year.

					Data representations - time-series, clustering (Agglomerative hierarchical)		
Tamersoy, 2015 [103]	Nicotine & Alcohol Use	Reddit: (subreddits - StopSmoking and StopDrinking)	Total – 86,835 posts and 766,574 comments from SS<br><br>59,201 posts and 492,573 comments from SD<br><br>(November 2014)	Understand how language and peer interactions on social media platforms can be leveraged in order to characterize the attributes of long-term abstinence from smoking or alcohol use. Identify key linguistic and interaction characteristics of short-term and long-term abstainers. Predict if short-term abstainers will proceed to long-term abstainer or will relapse	Supervised learning-based classification model – Ridge Regression<br><br>Response variable: if the user if short-term or long-term abstainer from smoking/drinking<br><br>Explanatory variables -<br><br>Language variables (n=304):<br>Feature Extraction – unigrams, bigrams and trigrams using bag-of-words model<br>Sentiment Analysis – VADER<br><br>Addiction variables (n=5): using addiction-related lexicons for smoking and drinking – Urban dictionary	Graph-centric metrics used such as indegree, outdegree, degree, reciprocity, clustering coefficient, #triangle, betweenness closeness and eigenvector centralities<br>Classification metrics - F1 score, RC, PR, ACC, specificity, AUROC	The best performing model was based on all three explanatory variables which were Language + Addiction + Interaction and it achieved F1 score of 0.86 for both the subcommunities. The results of the study showed that language and interaction between individuals on social media platforms can help in characterizing addiction-related health outcomes.

					Interaction variables - (based on activity measures, participation in other subreddits, and social network graph measures)<br>(10-fold cross validation)		

Abbreviations

ACC: accuracy
AMT: Amazon Mechanical Turk
API: application programing interface
AUROC: area under receiver operating characteristics curve
BiLSTM: bidirectional long short-term memory
CNN: convolutional neural network
DT: decision trees
GEE: generalized estimating equation
GloVe: global vectors
HTS: Hookah tobacco smoking
ISEAR: International Survey on Emotion Antecedents and Reactions
KNN: K-nearest neighbors
LDA: latent Dirichlet allocation
LIWC linguistic inquiry word count
LR: logistic regression
LSA: latent semantic analysis
LSTM: long short-term memory
ML: machine learning
NMUPD: non-medical usage of prescription drugs
PR: precision
RC: recall

RF: random forest
RI: random indexing
SemEval: semantic evaluation
SentiWords: sentiment words
SNA: social network analysis
SVM: support vector machine
TASA: Touchstone Applied Science Associates
TF-IDF: term frequency-inverse document frequency
VADER: Valence Aware Dictionary and sEntiment Reasoning
